# Mechanistic insights into the effect of phosphorylation on Ras conformational dynamics and its interactions with cell signaling proteins

**DOI:** 10.1016/j.csbj.2021.01.044

**Published:** 2021-02-09

**Authors:** Yuanhao Wang, Dong Ji, Chaoyu Lei, Yingfei Chen, Yuran Qiu, Xinyi Li, Mingyu Li, Duan Ni, Jun Pu, Jian Zhang, Qiang Fu, Yaqin Liu, Shaoyong Lu

**Affiliations:** a Department of Pathophysiology, Key Laboratory of Cell Differentiation and Apoptosis of Chinese Ministry of Education, Shanghai Jiao Tong University, School of Medicine, Shanghai 200025, China; b Department of Anesthesiology, Changhai Hospital, The Second Military Medical University, Shanghai 200433, China; c The Charles Perkins Centre, University of Sydney, Sydney, NSW 2006, Australia; d Department of Cardiology, Renji Hospital, Shanghai Jiao Tong University, School of Medicine, Shanghai 200120, China; e Medicinal Chemistry and Bioinformatics Centre, Shanghai Jiao Tong University, School of Medicine, Shanghai 200025, China; f Department of Orthopedics, Shanghai General Hospital, Shanghai Jiao Tong University, School of Medicine, Shanghai 200080, China

**Keywords:** K-Ras, Phosphorylation, Molecular dynamics simulations, Protein-protein interactions, Drug discovery

## Abstract

Ras undergoes interconversion between the active GTP-bound state and the inactive GDP-bound state. This GTPase cycle, which controls the activities of Ras, is accelerated by Ras GTPase-activating proteins (GAPs) and guanine nucleotide exchange factors (SOS). Oncogenic Ras mutations could affect the GTPase cycle and impair Ras functions. Additionally, Src-induced K-Ras Y32/64 dual phosphorylation has been reported to disrupt GTPase cycle and hinder Ras downstream signaling. However, the underlying mechanisms remain unclear. To address this, we performed molecular dynamics simulations (~30 μs in total) on unphosphorylated and phosphorylated K-Ras4B in GTP- and GDP-bound states, and on their complexes with GTPase cycle regulators (GAP and SOS) and the effector protein Raf. We found that K-Ras4B dual phosphorylation mainly alters the conformation at the nucleotide binding site and creates disorder at the catalytic site, resulting in the enlargement of GDP binding pocket and the retard of Ras-GTP intrinsic hydrolysis. We observed phosphorylation-induced shift in the distribution of Ras-GTP inactive-active sub-states and recognized potential druggable pockets in the phosphorylated Ras-GTP. Moreover, decreased catalytic competence or signal delivery abilities due to reduced binding affinities and/or distorted catalytic conformations of GAP, SOS and Raf were observed. In addition, the allosteric pathway from Ras/Raf interface to the distal Raf L4 loop was compromised by Ras phosphorylation. These results reveal the mechanisms by which phosphorylation influences the intrinsic or GAP/SOS catalyzed transformations between GTP- and GDP-bound states of Ras and its signal transduction to Raf. Our findings project Ras phosphorylation as a target for cancer drug discovery.

## Graphical abstract

**Figure:**
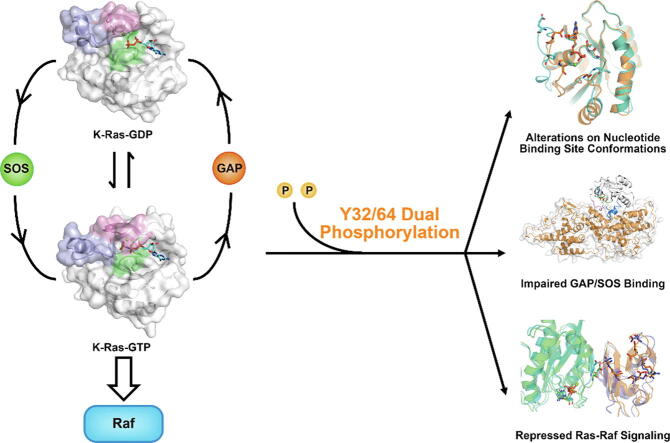

## 1 Introduction

Ras proteins are a group of small GTPases that play critical roles in intracellular signal transduction pathways that are essential for cell growth, proliferation, differentiation and survival [1], [2], [3]. It acts as a binary switch by existing in the GTP-bound active state and GDP-bound inactive state. The downstream signaling, such as Raf/MEK/ERK (MAPK) cascade and PI3K/Akt/mTOR cascade, is controlled by interconversion between these two states [4], [5], [6], [7]. Aberrant Ras mutations or post translational modifications lead to the dysfunction of ‘ON’-‘OFF’ control on Ras effectors, resulting in cancers as well as a collection of developmental conditions called “*RASopathies*” [3], [8], [9], [10]. It is estimated that oncogenic Ras mutations account for approximately 30% of human cancers and induce over 1 million deaths per year worldwide [3], [8]. There are four isoforms of Ras in human cells: H-Ras, N-Ras, K-Ras4A and K-Ras4B (splice variants of gene *KRAS*) [11]. Among them, K-Ras is the most frequently mutated isoform in human Ras-driven cancers (~85%, according to COSMIC), followed by N-Ras (~12%) and H-Ras (~3%) [12].

**Fig. 1:**
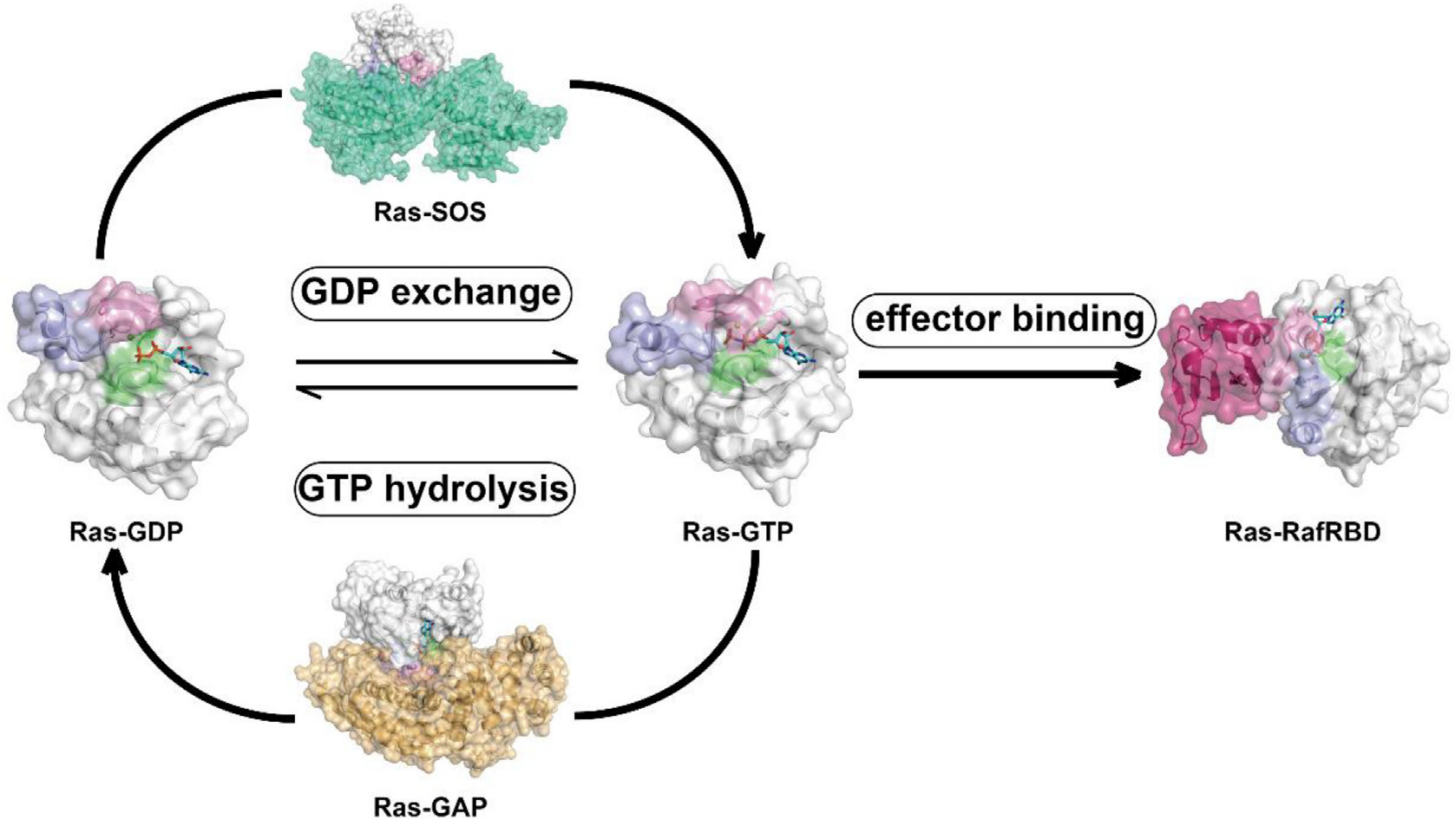
Ras GTPase cycle. Ras interconverts between active GTP-bound state (Ras-GTP) and inactive GDP-bound state (Ras-GDP). Ras regulatory proteins, known as SOS and GAP, facilitate the transformation between the two states. The most significant conformational differences between GDP-bound Ras and GTP-bound Ras are located at the nucleotide binding pocket, consisting of P-loop (lime), switch I (pink) and switch II (light blue) regions. In the GDP-bound state, the pocket is ‘open’, while in the GTP-bound state, it is ‘closed’. The GTP-bound Ras has higher affinity towards effector proteins (e.g., Raf), which keeps Ras signaling in the ‘ON’ state. (For interpretation of the references to colour in this figure legend, the reader is referred to the web version of this article.)

Ras activity status is regulated by the GTPase cycle, in which guanine nucleotide exchange activates GDP-bound Ras, and GTP hydrolysis deactivates GTP-bound Ras (Fig. 1) [4]. A couple of Ras regulatory proteins have been found to accelerate the conversions of Ras between the two states, assisting the realization of Ras’ switch function. GTPase-activating proteins (GAPs) stimulate the intrinsic GTP hydrolysis rate, thus facilitating the deactivation of Ras-GTP [13]. In contrast, Son of sevenless (SOS), a Ras-specific guanine nucleotide exchange factor (GEF), activates Ras by catalyzing the disassociation of GDP from Ras and the reloading of a GTP molecule [14]. Active GTP-bound Ras has high affinities towards effector proteins and can initiate downstream signals in the ‘ON’ state. On the contrary, the affinity of GDP-bound Ras to downstream cell signaling proteins is lesser than the GTP-bound Ras by several orders of magnitude, which effectively turns ‘OFF’ the Ras signaling. Ras-driven diseases occur when the GTPase cycle is not properly regulated, either because of the dysregulation of intrinsic- or GAP/SOS catalyzed-Ras GTP hydrolysis/guanine nucleotide exchange [4], [8], [15].

Structural insights into Ras proteins and their complexes with other cell signaling proteins such as Raf and PI3K can help us to understand the mechanisms underlying the Ras-driven cancers and to develop anti-Ras drugs for cancer therapies [14], [16], [17], [18], [19]. Conformations of three functional regions in the effector lobe (residues 1–87) of Ras, P-loop (residues 10–17), switch I (residues 32–38) and switch II (residues 59–76), are pivotal for Ras functions [4]. In the GTP-bound state, these three regions adopt a ‘closed’ conformation in the nucleotide binding site and are favorable for regulator/effector binding. Following GTP hydrolysis, the two switch regions as well as P-loop are rearranged into an ‘open’ conformation and are less suitable for interactions with Ras-related proteins. The overwhelming majority of oncogenic Ras mutations that hinder the formation of the ‘correct’ conformation for Ras functioning are located in the three regions, namely G12 (~89%), G13 (~9%), and Q61(~1%) [20], [17].

Earlier, Ras was considered “undruggable” due to its picomolar affinities towards GDP/GTP substrates and the lack of suitable allosteric pocket for small molecule ligands [19], [21]. However, unravelling of a large number of Ras structures and development of computational methods for protein structural investigations, in the last decade, have led to enormous breakthroughs in our understanding of Ras conformational dynamics and Ras-regulator/effector protein–protein interactions (PPIs) [14], [22], [23], [24], [25]. These developments have renewed structure-based drug design strategies [26], [27], [28], such as identification of cryptic allosteric sites in Ras and inhibition of Ras complexation with regulators or effectors [29], [30]. Attempts at targeting the ‘undruggable’ Ras have yielded several preliminary successes so far. For example, a K-Ras^G12C^ mutant inhibitor AMG510 that covalently binds to Ras cryptic allosteric pocket has recently been proceeded to phase II clinical trials [31], [32], [33]. Small molecules that target proteins’ interfaces between Ras and its regulators/effectors, such as Sulindac and DCAI, have been shown to inhibit Ras–Raf and Ras–SOS interactions [19], respectively. Nonetheless, the high mutant specificity or lack of potency of these compounds are major hurdles for their applications in cancer therapies, calling for further optimization and investigations.

Recently, Kano et al. reported that the Src- and SHP2- mediated K-Ras phosphorylation and dephosphorylation affect Ras downstream signaling by regulating the GTPase cycle [34]. Ras phosphorylation by Src halts the GTPase cycle and hinders the binding of Ras to Raf, while dephosphorylation by SHP2 re-activates Ras. The two phosphorylation sites Y32 and Y64, which are located at the respective switch I and II regions, constitute a major part of the guanosine nucleotide-binding pocket and occur at the interfacial areas in Ras complexes with regulators GAP/SOS as well as the effector Raf. However, the underlying mechanism this phosphorylation mediated inhibition of Ras transformation between GTP-bound and GDP-bound states and Ras interactions with Raf remains unclear. More importantly, as Ras point mutations produce novel sites for drug design, structural perturbations induced by phosphorylation could also alter Ras conformational dynamics and generate potential druggable pockets. Since Y32/Y64 are well conserved in human Ras proteins [35], targeting Src-phosphorylated Ras itself as well as its complexes with regulator and effector proteins is an appealing strategy to develop specific Ras inhibitors to treat Ras-driven cancers.

In this study, we performed explicit molecular dynamics (MD) simulations on multiple microsecond timescales, and investigated the effect of phosphorylation on K-Ras4B conformational dynamics and the PPIs with its regulators and effectors. We focused on the dynamic conformational changes induced by dual phosphorylation of Y32 and Y64, encompassing Ras in the GTP- and GDP-bound states, its complexes with GTPase cycle regulators GAP and SOS, as well as its complex with the effector protein Raf. From both structural and energetic aspects, we revealed the dual phosphorylation effects on Ras conformational transitions, binding affinities towards regulatory/effector proteins, and the stabilization of catalysis-active conformations. This study aims to shed light on the mechanistic understanding by which dual phosphorylation affected the equilibrium of Ras GTPase cycle and weakened downstream Raf signaling, inspiring further research on targeting phosphorylated Ras for cancer therapy.

## 2 Materials and methods

### 2.1 Construction of initial simulated systems

**a. GTP-bound K-Ras4B.** The crystal structure of GppNHp-bound K-Ras4B^Q61H^ (PDB ID 3GFT) was selected as the initial structure to model wild-type unphosphorylated and phosphorylated GTP-bound K-Ras4B. H61 was mutated back to Q using Discovery Studio 2016. GppNHp was replaced by a GTP molecule. For the phosphorylated system, both Y32 and Y64 were mutated to phosphotyrosine.

**b. GDP-bound K-Ras4B.** The crystal structure of GDP-bound K-Ras^WT^ (PDB ID 4LPK) [31] was selected as the initial structure for modeling wild-type unphosphorylated and phosphorylated GTP-bound K-Ras4B. The calcium atom in the GDP binding pocket was replaced by a magnesium atom. For the phosphorylated system, both Y32 and Y64 were mutated to phosphotyrosine.

**c. GTP-bound K-Ras4B–GAP system.** The crystal structure of GDP/AlF_3_-bound H-Ras^WT^ in complex with GAP (PDB ID 1WQ1) [36] was selected as the initial structure for wild-type unphosphorylated and phosphorylated K-Ras4B–GAP complexes. Mutations Q95H, D107E, A121P, A122S, E126D, S127T, R128K, Y141F, E153D, and Q165K were performed on H-Ras to translate into wild-type K-Ras4B, and the GDP/AlF_3_ was replaced by a GTP molecule and a magnesium atom. For the phosphorylated system, both Y32 and Y64 were mutated to phosphotyrosine. Disulfide bonds between GAP C771 and C876 were defined.

**d. GDP-bound K-Ras4B–SOS system.** The crystal structure of the SOS1 catalytic domain in complex with apo K-Ras4B^G12C^ (PDB ID 6EPL) [37] was selected as the initial structure of SOS. The crystal structure of GDP-bound K-Ras4B^WT^ (PDB ID 4LPK) [31] was selected as the initial structure of GDP-bound K-Ras4B. First, the missing residues were added using Discovery Studio 2016. Then, using ZDOCK Server [38], the GDP-bound K-Ras4B was docked to SOS^cat^ in 6EPL and the top 1 docking pose was selected. After that, 10,000 steps of minimization were performed using Discovery Studio 2016 using the steepest descent algorithm to optimize the docked interface. For the phosphorylated system, both Y32 and Y64 were mutated to phosphotyrosine.

**e. GTP-bound K-Ras4B–Raf system.** The crystal structure of GppNHp-bound H-Ras^WT^ in complex with RafRBD (PDB ID 4G0N) [39] was selected as the initial structure for both unphosphorylated and phosphorylated Ras–Raf complexes. GppNHp-bound K-Ras^Q61H^ was aligned to H-Ras^WT^ in 4G0N and H61 was mutated back to Q using Discovery Studio 2016. GppNHp was replaced by a GTP molecule. For the phosphorylated system, both Y32 and Y64 were mutated to phosphotyrosine.

### 2.2 MD simulations

A total of 10 systems were carried out using MD simulations, including unphosphorylated and phosphorylated GTP-bound K-Ras4B, GDP-bound K-Ras4B, GTP-bound K-Ras4B in complex with GAP, GDP-bound K-Ras4B in complex with SOS and GTP-bound K-Ras4B in complex with Raf. First, the initial parameter files for minimization and simulation were prepared using the Amber14 package with ff14SB force field [40] and general Amber force field (GAFF) [41]. The force field parameters for phosphorylated tyrosine were adopted in the phosphorylated systems [42]. Hydrogen atoms were added to each system using the Amber14 package. All systems were solvated to a truncated octahedron transferable intermolecular potential three point (TIP3P) water box [43], followed by the addition of Na^+^ and Cl^−^ counterions to neutralize overall charges and simulate the normal saline environment. Second, for all systems two rounds of energy minimizations were performed, with all atoms of proteins and nucleotides restrained in the first round and without any constraints in the second round. Then, all systems were heated from 0 K to 300 K within 300 ps in a canonical ensemble (NVT), followed by 700 ps system equilibration runs in NVT. After all the preparations were completed, three independent rounds of 1 μs MD simulations were performed with random velocities for each system under isothermal isobaric (NPT) conditions. For all systems, the particle mesh Ewald (PME) method [44] was employed for long-range electrostatic interactions while a cutoff distance of 10 Å was applied for short-range electrostatic interactions as well as van der Waals interactions. Covalent bonds involving hydrogens were restricted using the SHAKE method [45].

### 2.3 Cluster analysis

Cluster analyses using an average-linkage algorithm were carried out to extract the most representative structures from certain groups of snapshots in the MD trajectories [46]. The snapshots were superimposed using all Cα atoms beforehand to eliminate the overall rotation and transition.

### 2.4 Molecular mechanics Poisson-Boltzmann surface area (MM/PBSA) calculations

The MM/PBSA plugin from MMPBSA.py in the Amber14 package was used to calculate the binding free energies between Ras and regulators or effectors in both unphosphorylated and phosphorylated systems [47]. The binding free energy $\Delta G_{\mathrm{binding}}$ is defined as the change in the total Gibbs free energy change upon binding:

(1) $$\Delta G_{\mathrm{binding}}=G_{\mathrm{complex}}-G_{\mathrm{ligand}}-G_{\mathrm{receptor}}$$

where the Gibbs free energy of each system is predominantly composed of molecular mechanical energy ($E_{\mathrm{MM}}$), solvation energy ($G_{\mathrm{solv}}$) and the entropic compartments (-TS):

(2) $$\Delta G_{\mathrm{binding}}={(E}_{\mathrm{MM},complex}-E_{\mathrm{MM},ligand}-E_{\mathrm{MM},receptor})+{(G}_{\mathrm{solv},complex}-G_{\mathrm{solv},ligand}-G_{\mathrm{solv},receptor})-\left({\mathrm{TS}}_{\mathrm{complex}}-{\mathrm{TS}}_{\mathrm{ligand}}-{\mathrm{TS}}_{\mathrm{receptor}}\right)$$

Therefore, $\Delta G_{\mathrm{binding}}$ could be calculated by the total molecular mechanical energy change ${\Delta E}_{\mathrm{MM}}$, the total solvation energy change $\Delta G_{\mathrm{solv}}$, and the solute entropic contribution -TΔS:

(3) $$\Delta G_{\mathrm{binding}}={\Delta E}_{\mathrm{MM}}+\Delta G_{\mathrm{solv}}-T\Delta S$$

${\Delta E}_{\mathrm{MM}}$ could be further decomposed into a van der Waals component ${\Delta E}_{\mathrm{vdW}}$, a electrostatic component ${\Delta E}_{\mathrm{ele}}$, and an internal component ${\Delta E}_{\mathrm{int}}$ consisting of bond, angle and torsional energies. ${\Delta E}_{\mathrm{int}}$ is always equal to zero in our calculations, since the same configurations of ligands and receptors were used for their respective unbound and bound states.

(4) $$\Delta E_{\mathrm{MM}}={\Delta E}_{\mathrm{vdW}}+\Delta E_{\mathrm{ele}}+\Delta E_{\mathrm{int}}$$

For the ${\Delta G}_{\mathrm{solv}}$ term in Eq. (3), we applied the Poisson-Boltzmann continuum solvent model for calculations, and it is divided into the polar part ${\Delta E}_{\mathrm{PB}}$ and the nonpolar part ${\Delta E}_{\mathrm{nonpolar}}$:

(5) $$\Delta G_{\mathrm{solv}}={\Delta E}_{\mathrm{PB}}+\Delta E_{\mathrm{nonpolar}}$$

The nonpolar component ${\Delta E}_{\mathrm{nonpolar}}$ was calculated according to the solvent-accessible surface area (SASA) using Eq. (6), where the surface tension parameter was set to 0.00542 kcal·mol^−1^·Å^−2^, and the solvation parameter was set to 0.92 kcal/mol.

(6) $$\Delta E_{\mathrm{nonpolar}}=\gamma SASA+b$$

The conformation entropy component (-TΔS) can be calculated by normal mode analysis with a quasi-harmonic model. Considering the similar overall structure mode between unphosphorylated and phosphorylated systems and the low RMSDs of the unphosphorylated and phosphorylated systems, we omitted the -TΔS term.

### 2.5 Dynamic cross-correlation matrix (DCCM) analysis

The DCCM of all protein Cα atoms was calculated to reflect the inter-residue correlations [48]. The cross-correlation coefficient *C_ij_* was calculated as:

(7) $$C_{i,j}=\frac{\langle (r_{i}-\langle r_{i}\rangle \rangle ∙\langle (r_{j}-\langle r_{j}\rangle \rangle }{\sqrt{{\langle (r_{i}-\langle r_{i}\rangle \rangle }^{2}∙{\langle (r_{j}-\langle r_{j}\rangle \rangle }^{2}}}$$

where *r_i_* and *r_j_* represents the positions of the *i*th and *j*th Cα atoms.

### 2.6 Dynamic network analysis

Using the NetworkView plugin in VMD [49], we calculated the community organizations of unphosphorylated and phosphorylated Ras–RafRBD complex based on the correlation coefficient matrix *C_ij_*. Each Cα atom was recognized as a node, and for nodes that stayed within a cut-off distance of 4.5 Å for at least 75% of the simulation time, edges were drawn between these nodes [50]. The edge connections between certain nodes were calculated using Eq. (8):

(8) $$d_{i,j}=-log\left(\left|C_{i,j}\right|\right)$$

where *i* and *j* represent the two nodes and *C_ij_* was calculated using Eq. (7). In addition, optimal pathways and suboptimal pathways within 20 Å to the optimal pathway were calculated using Floyd–Warshall algorithm [51].

## 3 Results

### 3.1 Dual phosphorylation of GTP-bound K-Ras4B alters the conformation at the nucleotide binding site and hinders GTP hydrolysis

**Fig. 2:**
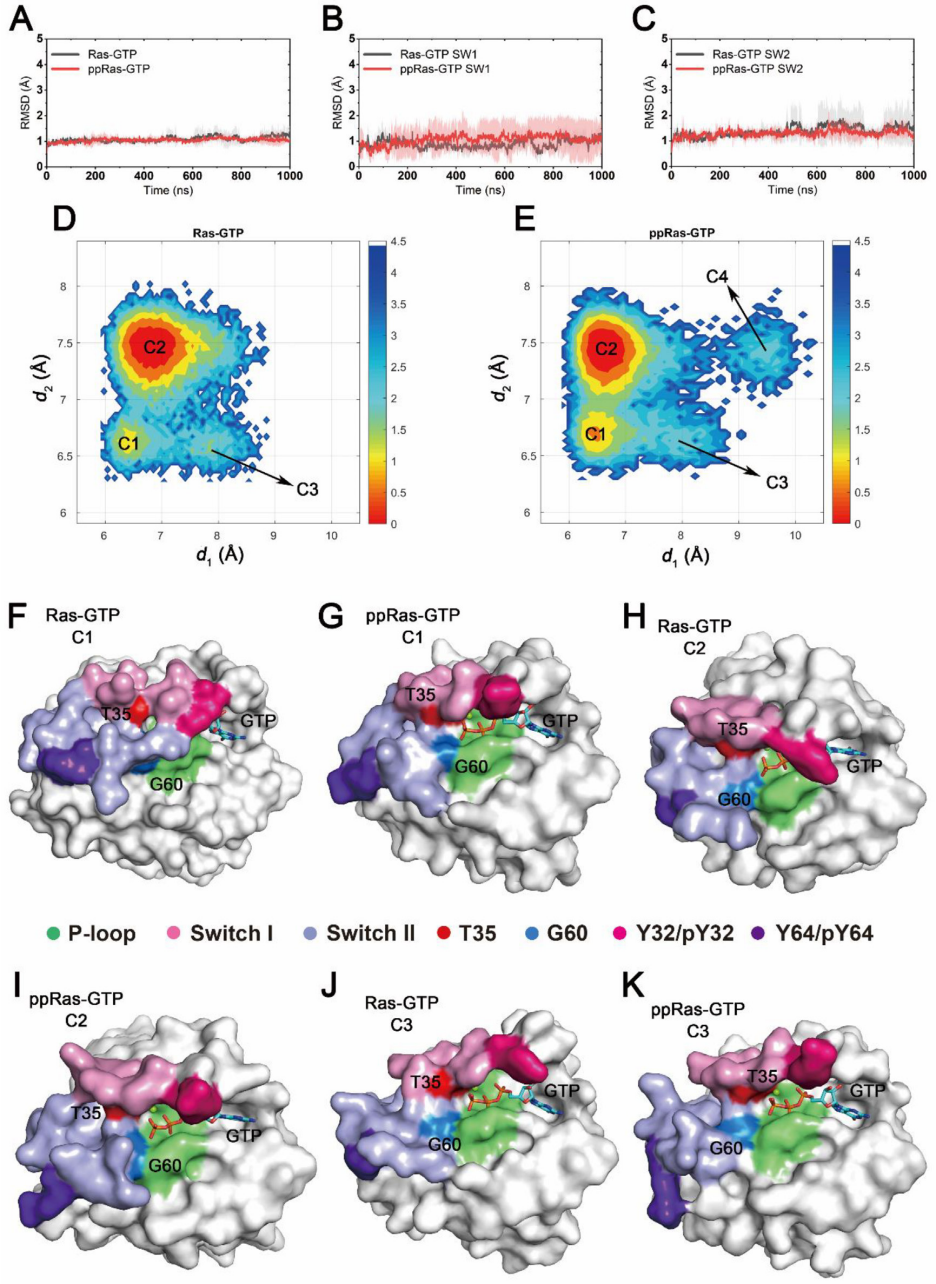
Conformational dynamics of unphosphorylated and phosphorylated GTP-bound K-Ras4B. RMSDs of Cα atoms in unphosphorylated (black) and phosphorylated (red) systems within (A) all Ras residues and (B) switch I residues and (C) switch II residues. Gray and pink transparencies represent the error. Conformational landscapes of unphosphorylated (D) and phosphorylated (E) K-Ras4B generated using *d*_1_ (distance from G60 Cα to GTP P_β_) and *d*_2_ (distance from T35 Cα to GTP P_β_). (F-K) Representative structures of conformational clusters C1–C3 in the two systems. K-Ras4B P-loop, switch I and switch II regions are colored lime, pink, and light blue, respectively. Surface of T35 and G60 residues are colored red and marine. (For interpretation of the references to colour in this figure legend, the reader is referred to the web version of this article.)

Three independent rounds of 1 μs MD simulations for both unphosphorylated and phosphorylated wild-type GTP-bound K-Ras4B alone were performed to capture the dynamic conformational changes induced by dual phosphorylation at Y32 and Y64. To compare the overall structural dynamics of the two systems, we calculated the root-mean-square deviations (RMSDs) of the C_α_ atoms relative to the initial crystal structure. As shown in Fig. 2A, both systems reached equilibrium soon after the simulations began. The RMSDs of unphosphorylated and Y32/Y64-phosphorylated K-Ras4B were 1.08 ± 0.18 Å and 1.05 ± 0.13 Å, respectively, suggesting no significant backbone conformational differences between the two systems during the simulations. In particular, we found that RMSDs of switch I and switch II in the phosphorylated form were similar to those of the unphosphorylated form (Fig. 2B-C). These results indicated that phosphorylation had only a minor effect on the overall backbone structure of GTP-bound K-Ras4B alone. Thus, we further investigated the detailed conformational dynamics of specific residues in functional switch I and switch II regions.

Based on MD simulations and NMR spectroscopic methods [17], [52], [53], it has been known that GTP-bound K-Ras4B exists in active and inactive states with discrepant conformations in the GTP-binding pocket. The relationship between GTP and the residues T35 in switch I and G60 in switch II reflects the most apparent structural distinctions between the active and inactive states [17]. In the active state of GTP-bound K-Ras4B, the two switch regions resemble the conformations found in Ras–effector complexes, with both G60 and T35 anchoring on to the two switch regions of Ras adjacent to GTP γ-phosphate [4], [31]. In contrast, three inactive sub-states of GTP-bound K-Ras4B, in which G60 and/or T35 residue(s) are partially or completely disassociated from GTP, are less suitable for effectors binding and dynamically interconvert into the active state. To study the active-inactive conversion equilibrium in both unphosphorylated and phosphorylated K-Ras4B, we projected the MD trajectories onto two-dimensional surface according to the distances from the G60 C_α_ atom to the GTP P_β_ atom (*d*_1_) and the T35 C_α_ atom to the GTP P_β_ atom (*d*_2_) (Fig. 2D-E). In the free-energy landscapes, conformations in each free energy basin with similar *d*_1_ and *d*_2_ values were categorized as clusters C1–C4. The distributions of the free-energy surface of the two systems were broadly similar, with a predominant basin (C1) and a sub-ordinate basin (C2) containing the majority of the simulation snapshots. Interestingly, energy sub-basins C3 and C4 were also observed in the unphosphorylated and/or phosphorylated systems, which indicated the detection of some infrequent conformations.

We extracted the representative structures of each cluster in the two systems using cluster analysis. As shown in Fig. S1A-S1B, representative structures of C1 were superimposed on the previously reported structure of active state H-Ras [54]. The relative locations and orientations of T35 and G60 in the two systems overlapped well with H-Ras, indicating that C1 represents the active state conformation. However, in the unphosphorylated K-Ras4B, the P-loop, switch I and switch II regions formed the ‘closed’ conformation of the GTP binding site, while in the phosphorylated system the GTP binding site was in a slightly ‘open’ conformation due to the outward orientation of pY32 (Fig. 2F-G and Fig. S1A-S1B). Since a close contact between Y32 side chain and GTP occurs in signaling-active Ras isoforms (e.g. H-Ras^G12D^, K-Ras4B^G12D^, RafRBD-bound H-Ras^WT^ and H-Ras^WT^) [55], the separation of pY32 form GTP phosphate group could result in decreased Ras signaling capabilities even though in its ‘active’ state. Within the predominant energy basins C2 of each system, the interaction between T35 and GTP was weakened, which is characteristic of the inactive form 2 [53]. Representative structures of C2 in both systems exhibited slightly alienated switch I, resulting in the opening of the GTP-binding site (Fig. 2H-I). Similarly, the side chain of Y32 was in the inward position in the unphosphorylated Ras, compared to the outward orientation in the phosphorylated Ras (Fig. S1C). In the C3 conformations, G60 was disassociated with GTP, resembling previously reported inactive form 3 structures (Fig. 2J-K and Fig. S1D). Furthermore, in the phosphorylated system we also observed a minor stable conformation C4, which has the characteristic structure of Ras inactive form 1 [56] (Fig. S1E). This distinct conformation is observed in all three independent replicas of simulations of the phosphorylated Ras-GTP, while no similar structures were detected in the unphosphorylated system. Representative structures of C4, presented in Fig. S2A-S2C shared comparable overall conformations between each individual run, further confirming that the observed C4 conformation was not derived from an accidental simulation bias. Taken together, Y32 and Y64 dual phosphorylation not only perturbed the distributions of active/inactive sub-states of GTP-bound K-Ras4B, but also altered Y32 side-chain orientations, causing conformational changes in the GTP binding site. This probably leads to dysfunctional active GTP-bound Ras, since Ras downstream signaling depends on the proper architecture of two switch regions, especially the Y32 orientation.

In addition, considering that previous studies have identified surface drug pockets in the wild-type and mutant H-Ras inactive form 1 (Fig. S3A) as well as K-Ras4B^WT^ inactive form 3 (corresponding to C4 and C3 in our study) [4], we also explored potential druggable pockets on the surface of dual phosphorylated K-Ras4B using fpocket software [57]. In phosphorylated Ras-GTP C4, a pocket that is mainly composed of switch II was detected (Fig. S3B). A preliminary evaluation of the druggability of the pocket was indicated by the fpocket druggability score of 0.731, suggesting a strong possibility for drug-like molecule binding. Similarly, in both unphosphorylated and phosphorylated Ras-GTP C3, we also discovered potential pockets, with druggability scores of 0.716 and 0.233, respectively (Fig. S3C-S3D). These observations may inspire future drug design research to target phosphorylated Ras for direct Ras inhibitors.

**Fig. 3:**
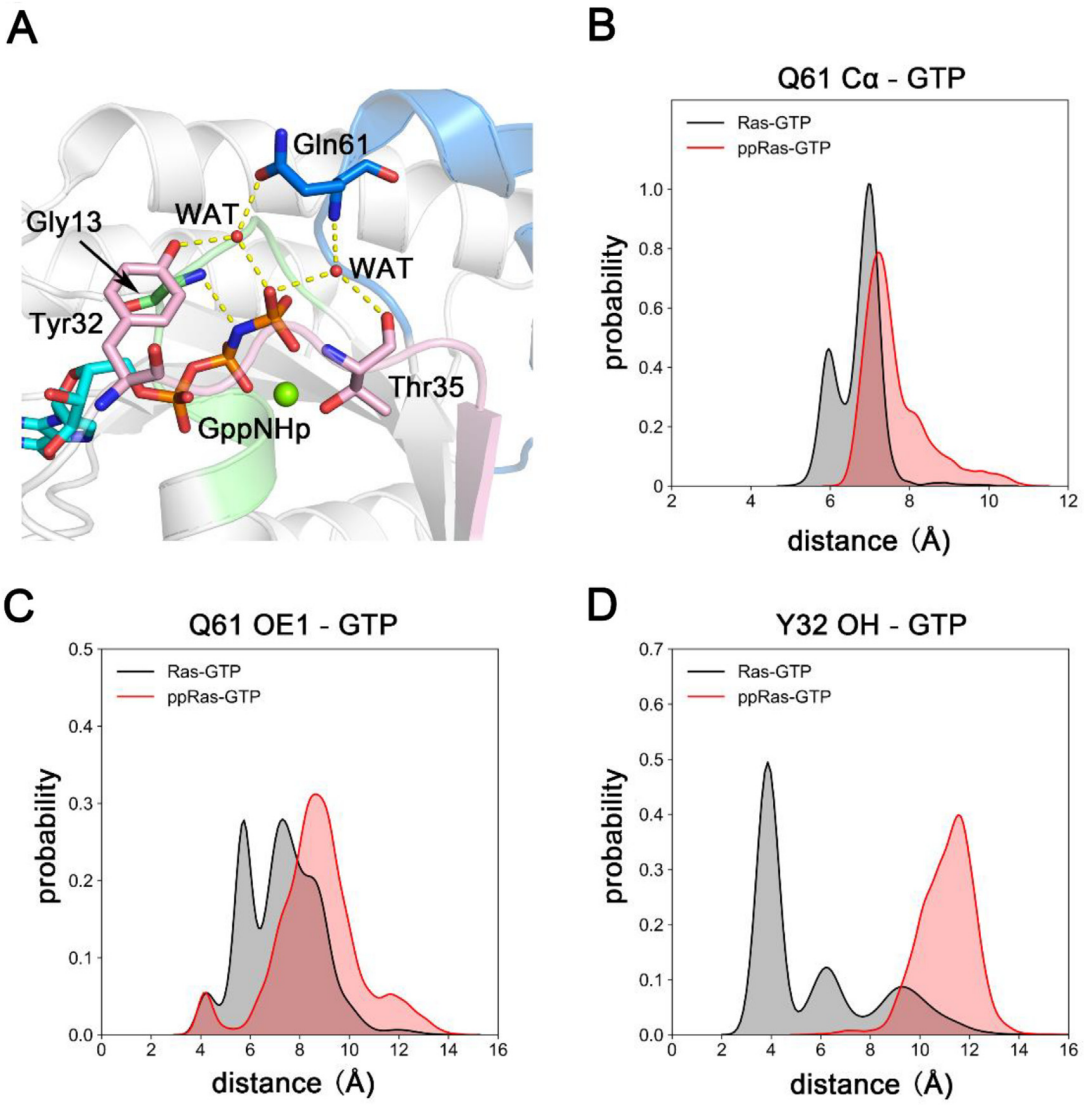
Ras GTP hydrolysis site. (A) Key residues responsible for the active conformation in the pre-catalytic state for GTP-hydrolysis in H-Ras (PDB ID 3K8Y). Pivotal inter-residue distance distributions reflecting K-Ras4B GTP-hydrolysis capability, (B) from Q61 Cα atom to GTP Pγ atom, (C) from Q61 OE1 atom to GTP Pγ atom and (D) from Y32/pY32 side chain OH atom to GTP Pγ atom.

On the other hand, we also concentrated on the GTPase catalytic center to explore the impact of dual phosphorylation on Ras intrinsic hydrolysis activity. Several proposed mechanisms for the intrinsic hydrolysis of GTP-bound Ras suggested that Q61 plays an important role in the pre-catalytic conformations [54], [58], [59], [60]. For reference, the hydrolysis-active conformation of GTP-bound H-Ras (PDB ID 3K8Y) [54] is shown in Fig. 3A. The side chains of Y32 in switch I and Q61 in switch II form hydrogen bonds with a bridging water molecule, which together with G13 neutralizes the accumulated negative charge on the β-γ bridging oxygen atom of GTP to facilitate the disassociation of the GDP leaving group from the γ-phosphate. The backbone N atom of Q61 was found to interact with a nucleophilic water molecule to stabilize an in-line attack towards GTP γ-phosphate. To compare the intrinsic hydrolysis activity of unphosphorylated and phosphorylated Ras, we measured three pair-wise distances throughout the trajectories. The probability distributions of the distances between Q61 C_α_ atom and GTP P_γ_ atom indicated that in the phosphorylated system, Q61 moved away from the active site for catalysis (Fig. 3B). Distances from the Q61 OE1 atom to the GTP P_γ_ atom and from the Y32 OH atom to the GTP P_γ_ atom suggested that upon phosphorylation, the mobilization function of the bridging water for hydrolysis was impaired due to the lack of H-bond formation (Fig. 3C-D). Starting from 3.8 Å, the distance between pY32 OH and GTP P_γ_ in the phosphorylated Ras soon reached to and stabilized at around ~12 Å (Fig. S4). This is mainly because the inter-attraction between the GTP phosphate group and the side chain of Y32 was replaced by the strong electrostatic repulsion between the negatively charged phosphate groups on the Y32 sidechain and GTP, causing significant outward motion of the Y32 side chain. The steric hindrance effect between the bulky phosphate group on pY32 and the narrow GTP-binding pocket may also be attributed to the re-orientation of the pY32 side chain.

In summary, dual phosphorylation of Y32 and Y64 impeded the stabilization of the catalytic-active conformation, thus inhibited GTP hydrolysis, which is in accordance with the experimental observations that phosphorylation stalls the intrinsic hydrolysis of GTP-bound K-Ras4B [34].

### 3.2 Dual phosphorylation of GDP-bound K-Ras4B expands Ras nucleotide binding pocket

**Fig. 4:**
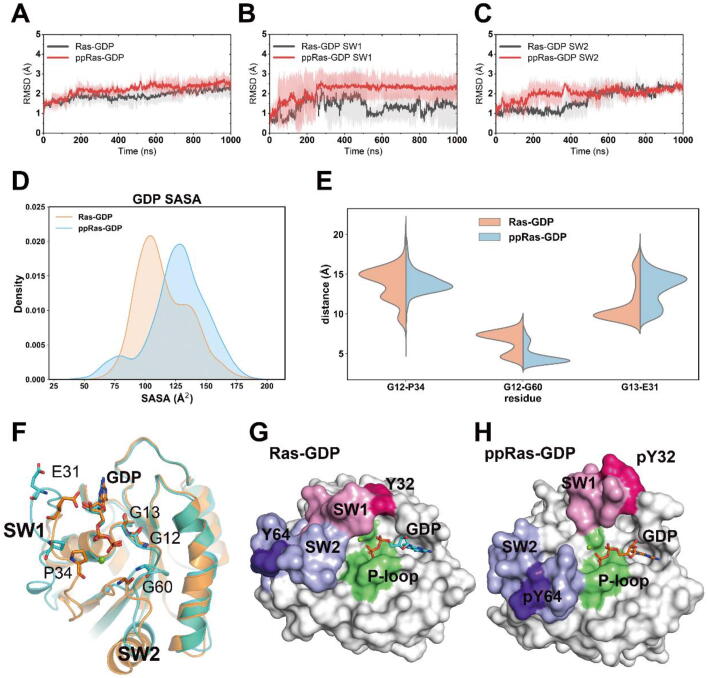
Conformational dynamics of unphosphorylated and phosphorylated GDP-bound K-Ras4B. RMSDs of Cα atoms in unphosphorylated (black) and phosphorylated (red) systems within (A) all Ras residues and (B) switch I residues and (C) switch II residues. Gray and pink transparencies represent the error. (D) Average SASAs of GDP. (E) Distance distributions of Cα atoms between G12-P34, G12-G60, and G13-E31. (F) Cartoon representations of GDP binding pocket in unphosphorylated (orange) and phosphorylated (cyan) K-Ras4B. Representative structures of the surface of the GDP binding pocket in the unphosphorylated (G) and phosphorylated (H) K-Ras4B. (For interpretation of the references to colour in this figure legend, the reader is referred to the web version of this article.)

In the GDP-bound state of K-Ras4B, the switch I and switch II regions exhibit significant conformational differences in comparison with the GTP-bound state [61]. To explore the influence of dual Y32 and Y64 phosphorylation on GDP-bound K-Ras4B conformational dynamics, we performed three independent rounds of 1 μs MD simulations of unphosphorylated and phosphorylated Ras-GDP. After ~200 ns simulations, both systems reached equilibrium with an average overall RMSD of 1.98 ± 0.38 Å and 2.33 ± 0.32 Å relative to the initial crystal structure (PDB ID 4LPK), respectively (Fig. 4A). Although only a modest distinction was noted between the whole backbone structures, the RMSD of switch I in the phosphorylated Ras was notably higher than that of the unphosphorylated Ras (Fig. 4B). The RMSDs of the switch II region, however, were similar in the two systems (Fig. 4C).

Through a nucleotide exchange mechanism, the inactive GDP-bound Ras disassociates with GDP and reloads a GTP molecule, transforming into the GTP-bound active state. This process is involved in the opening of the nucleotide binding pocket, which mainly consists of P-loop, switch I and switch II regions [4]. Previous experimental observations suggested that the dual phosphorylated form had a higher intrinsic nucleotide-exchange rate than the unphosphorylated form [34]. To determine the volume of the nucleotide binding site, we calculated the solvent-accessible surface-area (SASA) [62] of GDP (Fig. 4D). In the unphosphorylated system, the value of GDP SASA was 114.77 ± 21.49 Å^2^, while in the phosphorylated system the GDP SASA reached 126.54 ± 24.45 Å^2^, and the significance of this difference was confirmed by *t*-test (t = −32.35, p < 0.001). Further, we measured three pairs of inter-residue distances to evaluate the detailed contributions of each region of Ras towards the opening of the nucleotide binding pocket, and the probability distributions of these distances are shown in Fig. 4E. Distances between G12 Cα atom of P-loop and P34 Cα atom of switch I as well as between the G12 Cα atom of P-loop and the G60 Cα atom of switch II best describe the size of the GDP phosphate binding site. The average distance of G12-P34 showed no obvious distinctions between unphosphorylated and phosphorylated systems, while the G12-G60 distance was slightly reduced in the phosphorylated system. In contrast, there was an apparent increase in G13-E31 Cα atom distances in the phosphorylated Ras, which represents the GDP ribose binding site. The analysis of the three pair-wise distances suggested that the GDP binding pocket was expanded in the phosphorylated form, which was mainly attributed to the segregation of switch I and P-loop.

Furthermore, we superimposed the representative structures of the two systems extracted from the equilibrium stage of each run (Fig. 4F). Predominant conformational variations between unphosphorylated and phosphorylated Ras were found in the switch I and switch II regions. The switch I region of phosphorylated Ras distinctly stretched away from the P-loop to provide space for GDP disassociation, notwithstanding the minor approximation of switch II towards the P-loop (Fig. 4G-H). Detailed analysis of the representative structures of the two systems revealed that in phosphorylated Ras, the steric hindrance between the pY32 phosphate group and surrounding residues, especially Y40, caused the loss of the intra-molecular hydrogen bonds and the outward motion of switch I away from the nucleotide binding pocket (Fig. S5). In addition, electrostatic attraction between pY64 and R102 drags switch II towards helix α3, resulting in the departure of the two switch regions in Ras (Fig. S5). Collectively, these results suggested that dual Y32 and Y64 phosphorylation largely affected the conformation of the switch I region of GDP-bound K-Ras4B, thus amplifying the nucleotide binding pocket and increasing its intrinsic nucleotide-exchange rate.

### 3.3 Dual phosphorylation predominantly distorts the favored conformation of K-Ras4B–GAP’s for GTP-hydrolysis catalysis.

After exploring the conformational dynamics of phosphorylated GTP-bound and GDP-bound K-Ras4B, we explored the effect of phosphorylation on the PPIs with Ras regulator proteins GAP/SOS and Ras effector Raf, which are essential in the regulation of Ras signaling. We compared the interactions between GAP and Ras in the unphosphorylated system and that in the phosphorylated system to investigate the influence of phosphorylation on the GAP catalyzed GTP-hydrolysis process catalyzed by GAP.

**Fig. 5:**
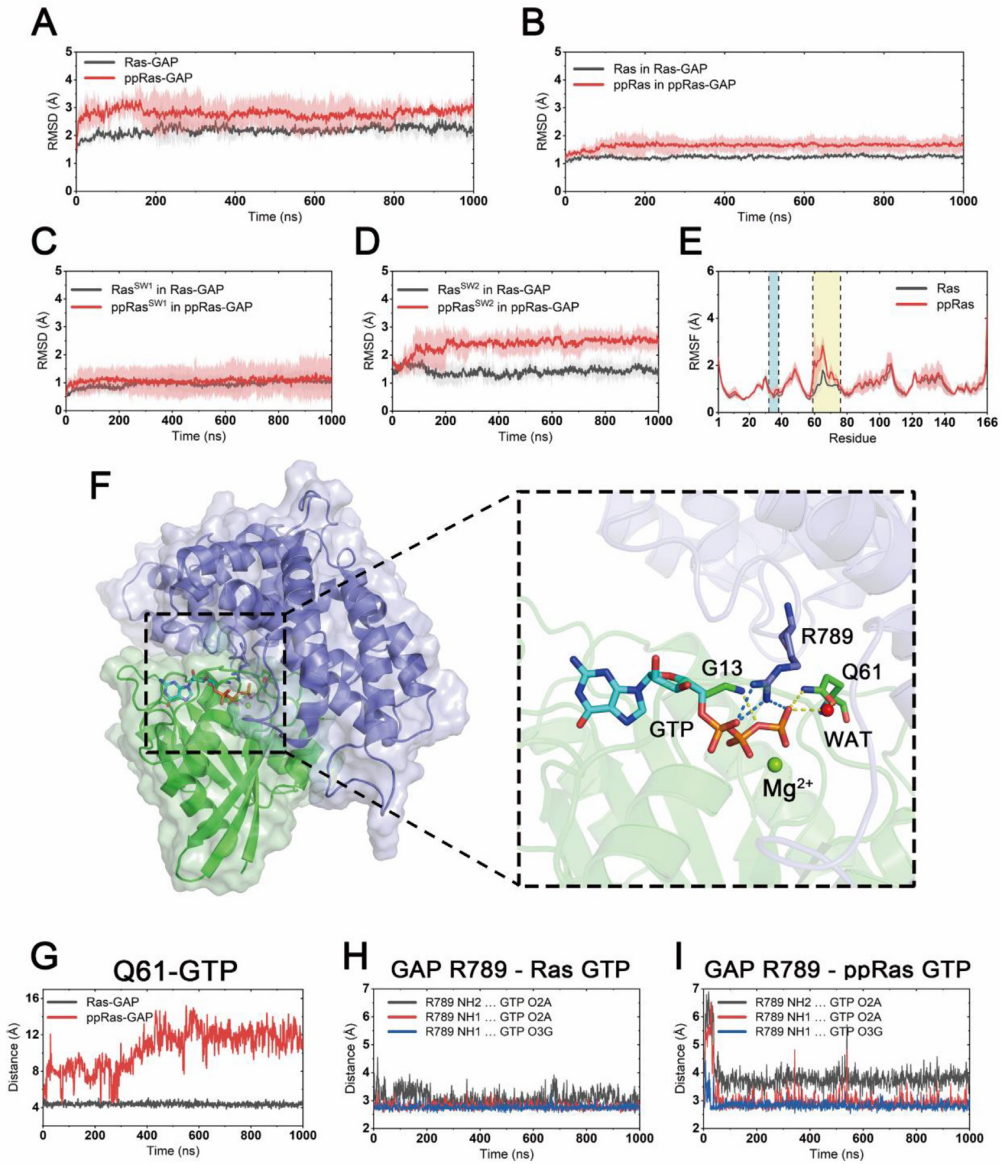
Conformational dynamics of unphosphorylated and phosphorylated GTP-bound K-Ras4B in complex with GAP. RMSDs of Cα atoms in unphosphorylated (black) and phosphorylated (red) systems within (A) all Ras and GAP residues; (B) all Ras residues; (C) Ras switch I residues and (D) Ras switch II residues. Gray and pink transparencies represent the error. (E) RMSF of Ras residues. The two switch regions are marked with blue and yellow backgrounds. (F) Representative structure of unphosphorylated K-Ras4B–GAP complex in a GTP-hydrolysis pre-catalytic conformation. (G) Distances from Ras Q61 side chain OE1 atom to GTP P_γ_ atom. Three atom-pair distances from GAP arginine finger R789 to GTP in the unphosphorylated system (H) and the phosphorylated system (I). (For interpretation of the references to colour in this figure legend, the reader is referred to the web version of this article.)

For both phosphorylated and unphosphorylated Ras, the GAP-bound complex approached equilibrium after ~200 ns MD simulations (Fig. 5A). The overall backbone RMSD for phosphorylated Ras relative to the start-point structure was higher than the unphosphorylated Ras (1.67 ± 0.22 Å versus 1.25 ± 0.09 Å), indicating some structural discrepancies between unphosphorylated and phosphorylated Ras (Fig. 5B). Focusing on the switch I and switch II regions, we found that the discrepancies mainly lay in the switch II region. (Fig. 5C-D). Meanwhile, root-mean-square fluctuation (RMSF) analysis revealed that phosphorylation induced greater switch II fluctuation, while little distinction was found in switch I region (Fig. 5E). This indicated a less constrained conformation of switch II in the phosphorylated K-Ras4B–GAP complex. With this evidence, we concentrated on Ras switch II to investigate the impact on the hydrolysis catalysis activity and GAP binding affinity upon phosphorylation.

As discussed above, by facilitating the nucleophilic water attacking the γ-phosphate on GTP, the Q61 residue in the switch II region plays an important role in the intrinsic GTP hydrolysis. Several studies on the structural mechanism of GAP-accelerated GTP hydrolysis concluded that GAP stabilizes the ‘correct’ arrangement of the catalytic Q61 of K-Ras4B and activates hydrolysis via its ‘arginine finger’ R789 [17], [63], [64]. Thus, the increase in switch II flexibility in the phosphorylated Ras suggested that the catalytic conformation of switch II was affected by phosphorylation, thereby impeding the GAP-accelerated Ras GTP hydrolysis. To confirm this notion, we extracted representative structures from the trajectories and found that the catalytic center of the unphosphorylated K-Ras4B–GAP complex was in a pre-catalytic conformation (Fig. 5F). Superseding the role of bridging water in the intrinsic hydrolysis mechanism, the arginine finger R789 of GAP protrudes into the GTP binding pocket and balances the negative charge on GTP phosphate groups. The side chain OE1 atom of Q61 was hence able to activate the nucleophilic water while at the same time formed a hydrogen-bond between Q61 NE2 atom and GTP γ-phosphate group. In this conformation the O–H bond in the nucleophilic water was polarized and enabled the hydroxyl group to attack GTP to initiate hydrolysis. GAP R789 as well as Ras G13/Q61 neutralized the developing negative charge on GTP in the transient state, thereby facilitating the decomposition of GDP and γ-phosphate.

According to the proposed mechanism for GAP-accelerated Ras GTP hydrolysis, we measured a series of interatomic distances and angles during our simulations to probe the strength of the interactions between GAP/Ras and GTP. The distance between the Ras Q61 CD atom and GTP P_γ_ atom evaluates the catalytic activities of Q61 for hydrolysis. As shown in Fig. 5G, in the unphosphorylated system, the side chain of Ras Q61 steadily adopted a close contact with GTP, which implied a catalytically active conformation. However, in the phosphorylated system, the Ras Q61 side chain was disordered. Analysis of the angle between Q61 NE1 and OE1 atoms as well as GTP P_γ_ atom also indicated that phosphorylation on Y32 and Y64 hampered the Q61 catalytic competence in the K-Ras4B–GAP complex (Fig. S6). Furthermore, using three atom-pair distances, we evaluated the neuralization effect of GTP negative charge by GAP, which is an indication of its hydrolytic activity (Fig. 5H-I). In the unphosphorylated system, the guanidine N atoms of GAP R789 were firmly connected to the electronegative oxygens on GTP α- and γ-phosphates, while a greater precariousness of these interactions was observed in the phosphorylated system. Together, these results suggest that the catalytic conformation was markedly disordered in the phosphorylated K-Ras4B–GAP complex. Thus, it was confirmed that dual Y32 and Y64 phosphorylation significantly hindered GAP-accelerated Ras GTP hydrolysis.

We next explored the impact of phosphorylation on the binding affinities between GTP-bound K-Ras4B and GAP. Based on molecular mechanics Poisson-Boltzmann surface area (MM/PBSA), we calculated the Gibbs free energies of the binding process ($\Delta G_{\mathrm{binding}}$) between K-Ras4B and GAP (Table 1). The calculated binding free energies are −136.7076 ± 9.5566 kcal/mol and −132.5768 ± 11.5709 kcal/mol for unphosphorylated and dual phosphorylated systems, respectively. Although the affinity of phosphorylated K-Ras4B towards GAP was reduced by an average of ~4 kcal/mol, the difference between the two systems is not statistically significant. Detailed examination of the energy contributions suggests that the slightly decreased binding affinity for the phosphorylated system was mainly due to the elevated electrostatic solvation energies, which is most probably resulted from the two highly negatively charged phospho-tyrosine groups.

**Table 1:** Binding free energy (kcal/mol) analysis between Ras and GAP.*

	Ras-GAP	ppRas-GAP
$\Delta E_{\mathrm{vdw}}$^a^	−129.7694 (9.0771)	−132.7643 (9.6633)
$\Delta E_{\mathrm{ele}}$^b^	−108.5026 (15.8423)	−135.7027 (24.4780)
$\Delta E_{\mathrm{PB}}$^c^	116.4448 (13.9793)	153.3894 (21.8991)
$\Delta E_{\mathrm{nonpolar}}$^d^	−14.8804 (0.8514)	−17.4992 (0.6586)
$\Delta E_{\mathrm{MM}}$^e^	−238.2721 (18.3031)	−268.4670 (28.4753)
$\Delta G_{\mathrm{solv}}$^f^	101.5644 (13.8258)	135.8902 (21.5386)
$\Delta G_{binding}$	−**136.7076 (9.5566)**	−**132.5768 (11.5709)**

*a. The van der Waals component and *b*. the electrostatic component in the change of the total molecular mechanical energy of the Ras-GAP complex. *c.* The electrostatic component determined by the Poisson Boltzmann (PB) equation. *d*. The nonpolar component determined by the solvent-accessible surface term in the change of the solvation free energy. *e.* Total molecular mechanical energy change and *f.* total solvation energy change. All numbers in parentheses represent standard deviations.

In summary, Ras Y32/64 dual phosphorylation significantly disturbs the organization of a suitable conformation for GAP-catalyzed GTP hydrolysis, blocking the conversion of phosphorylated Ras from the GTP-bound state to the inactive GDP-bound state. Ras–GAP binding energy factor may also play a role in the impaired GAP activity, but under our simulation timescale no predominant differences on the $\Delta G_{\mathrm{binding}}$ were found.

### 3.4 Dual phosphorylation blocks SOS binding and impedes GDP disassociation

The SOS-catalyzed transformation from Ras-GDP to Ras-GTP follows a multi-step mechanism [65], [66], [67]. First, GDP-bound Ras forms a low-affinity docking complex with SOS [68], followed by the opening of the Ras nucleotide binding site and the disassociation of Ras and GDP molecules [66]. The intermediate complex, apo Ras–SOS, then incorporates a GTP molecule and enables Ras-GTP to disassociate with SOS. To date, structural insights into the interactions between the SOS catalytic domain (hereafter referred to as SOS^cat^) and Ras are mostly engaged in the intermediate complex apo Ras–SOS, in which Ras and SOS are tightly combined [4]. However, the structural dynamics of Ras–SOS ‘recognition’ process, when GDP-bound Ras identifies and attaches to SOS^cat^, have not been well explored. Therefore, for a better understanding of the binding and activating processes of GDP-bound K-Ras4B and SOS, and to reveal the effect of dual Y32 and Y64 phosphorylation on this process, we established models of both unphosphorylated and phosphorylated GDP-bound K-Ras4B in complex with SOS^cat^ using the ZDOCK server [38].

**Fig. 6:**
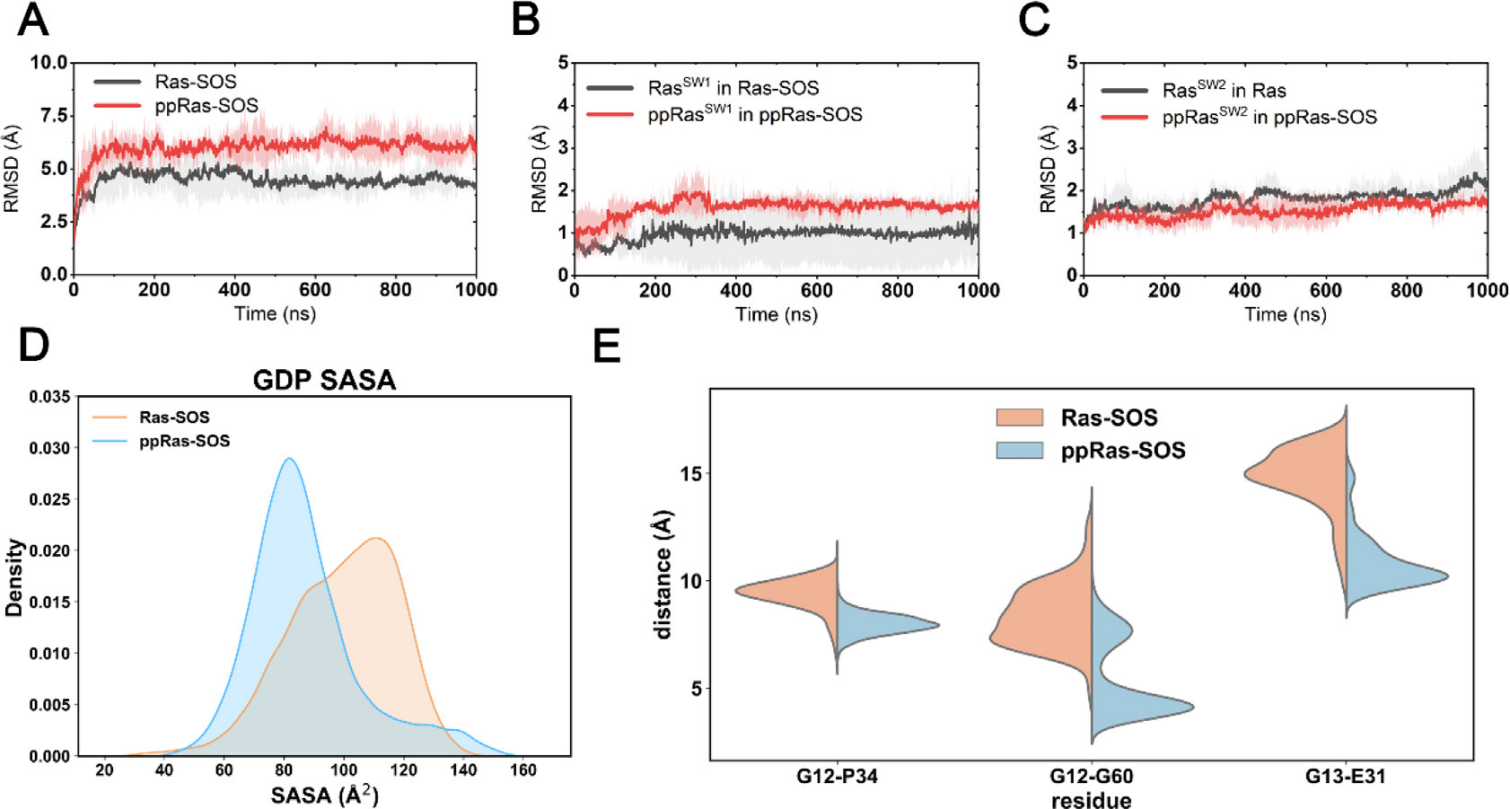
Conformational dynamics of unphosphorylated and phosphorylated GDP-bound K-Ras4B in complex with SOS. RMSDs of Cα atoms in unphosphorylated (black) and phosphorylated (red) systems within (A) all Ras and SOS residues; (B) Ras switch I residues, and (C) Ras switch II residues. Gray and pink transparencies represent the error. (D) Average SASAs of GDP. (E) Distance distributions of Cα atoms between G12-P34, G12-G60, and G13-E31 residues. (For interpretation of the references to colour in this figure legend, the reader is referred to the web version of this article.)

In the docked complex, the GDP-bound Ras was found to be similar in positions and orientations to the nucleotide-free Ras in the original crystal structure (PDB ID 6EPL) (Fig. S7A-S7B). Consistent with previous experimental observations [66], the switch II of GDP-bound Ras in the docked complex was also buried in the bowl-shaped depression region of SOS^cat^, holding Ras tightly towards SOS. Moreover, the switch I region formed interactions with the αH helix of SOS, which is vital to the catalytic functions of SOS for GDP disassociation [4]. Further analysis of protein interfacial interactions using PISA (Proteins, Interfaces, Structures and Assemblies) [69], [70] also confirmed that in our docked systems, GDP-bound K-Ras4B and SOS^cat^ adopted similar interfacial areas to the nucleotide-free Ras–SOS complex (Fig. S7C-S7D) [37]. Thus, using the docked complexes as the initial structure, we performed three rounds of 1 μs MD simulations on both the unphosphorylated and phosphorylated systems. The RMSDs of both systems reached equilibrium after ~100 ns simulations (Fig. 6A), implying that each system had attained a relatively stable conformation. Insights into the RMSDs of the two switch regions, we observed no predominant differences between the two systems (Fig. 6B-C). Therefore, we moved on to energetic investigations on how differences in these regions influence Ras–SOS binding.

**Fig. 7:**
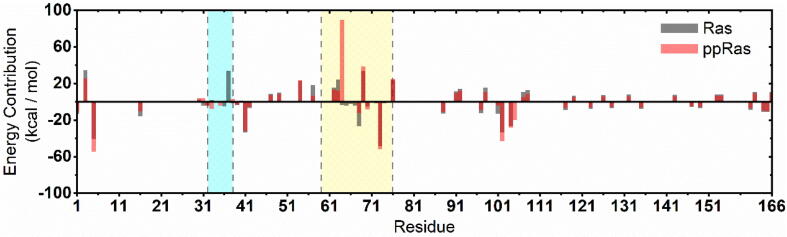
Binding free energy decomposition of the residues of Ras in K-Ras4B–SOS complex. Switch I and switch II regions of K-Ras4B are marked with blue and yellow backgrounds, respectively. (For interpretation of the references to colour in this figure legend, the reader is referred to the web version of this article.)

To quantify the effect of phosphorylation on Ras–SOS-binding free energies, we performed MM/PBSA calculations. As listed in Table 2, phosphorylation exerted an apparent binding free energy decrease (~23 kcal/mol) between K-Ras4B and SOS^cat^. This indicated an energetically unfavored binding process of phosphorylated K-Ras4B and SOS^cat^, therefore down-regulating the SOS-catalyzed Ras activation. We further decomposed $\Delta G_{\mathrm{binding}}$ into each residue in K-Ras4B to investigate the energy contributions for SOS binding (Fig. 7). As expected, the interfacial residues in K-Ras4B switch I, switch II, and L7 loop regions mainly contributed to the binding process. Notably, in contrast to the unphosphorylated K-Ras4B, the switch II region in the phosphorylated form strongly rejected SOS binding, especially the phosphorylated Y64. In addition, PISA analyses of representative structures of each system also confirmed the notion that phosphorylation of Ras significantly impaired SOS binding, since fewer inter-molecular hydrogen bonds and salt bridges were detected at the interface (Table S1 and Table S2).

**Table 2:** Binding free energy (kcal/mol) analysis between Ras and SOS.*

	Ras-SOS	ppRas-SOS
$\Delta E_{\mathrm{vdw}}$^a^	−104.9901 (17.4764)	−72.3901 (7.1425)
$\Delta E_{\mathrm{ele}}$^b^	4.7277 (21.8057)	11.9305 (24.8713)
$\Delta E_{\mathrm{PB}}$^c^	25.4543 (19.4151)	6.5030 (22.0907)
$\Delta E_{\mathrm{nonpolar}}$^d^	−12.4001 (1.5004)	−10.2631 (0.5994)
$\Delta E_{\mathrm{MM}}$^e^	−100.2624 (25.9905)	−60.4595 (26.5680)
$\Delta G_{\mathrm{solv}}$^f^	13.0542 (19.0815)	−3.7601 (21.8600)
$\Delta G_{binding}$	−**87.2082 (13.4710)**	−**64.2197 (7.6986)**

*a. The van der Waals component and *b*. the electrostatic component in the change of the total molecular mechanical energy of the Ras–SOS complex. *c.* The electrostatic component determined by the Poisson Boltzmann (PB) equation. *d*. The nonpolar component determined by the solvent-accessible surface term in the change of the solvation free energy. *e.* Total molecular mechanical energy change and *f.* total solvation energy change. All numbers in parentheses represent standard deviations.

Moreover, by analyzing the structural dynamics of the nucleotide binding site, we found that phosphorylation shrank the GDP pocket in the K-Ras4B–SOS complex. The corresponding GDP SASAs in the unphosphorylated and phosphorylated systems were 99.62 ± 18.15 Å^2^ and 87.09 ± 18.59 Å^2^, respectively (Fig. 6D). The *t*-test suggested that the difference between the two systems was significant (t = 43.13, p < 0.05). This indicated a lower possibility for GDP in the phosphorylated system to solvate and to disassociate with K-Ras4B. Furthermore, three pairs of inter-residue distances in K-Ras4B (Cα atoms of G12-P34, G12-G60 and G13-E31) were calculated to discern the detailed contributions for the contraction of the GDP binding pocket. As shown in Fig. 6E, Ras GDP nucleotide binding site (measured by distances of G12-P34 and G12-G60 pairs) as well as the ribose binding site (measured by distance of G13-E31 pair) were markedly constrained in the phosphorylated form. The larger GDP binding pocket in the unphosphorylated Ras-SOS complex also accounts for a higher GDP exchange rate observed experimentally [34]. In summary, our results revealed that dual phosphorylation not only caused a significant reduction in binding affinity between GDP-bound K-Ras4B and SOS, but also contracted the GDP binding pocket. Thus, GDP-bound K-Ras4B is less likely to interact with SOS^cat^, and the opening of the GDP pocket upon binding to SOS was restricted, resulting in the obstructed SOS-catalyzed activation of Ras.

### 3.5 Dual phosphorylation represses allosteric regulations towards RafRBD

GTP-bound Ras transmits activation signaling to the Raf and downstream MAPK pathway. Current understanding of Ras–Raf interactions suggests that Ras switch I and switch II interact with the Ras-binding domain (RafRBD) and cystine-rich domain (RafCRD) of Raf, respectively [4]. Since the mechanism of Ras/RafCRD interactions remained unresolved, and the binding affinity between Ras and RafRBD significantly surpasses that of Ras and RafCRD, we constructed unphosphorylated and phosphorylated complexes of GTP-bound K-Ras4B and RafRBD to investigate how Ras phosphorylation influences its interactions with the effector protein Raf.

**Fig. 8:**
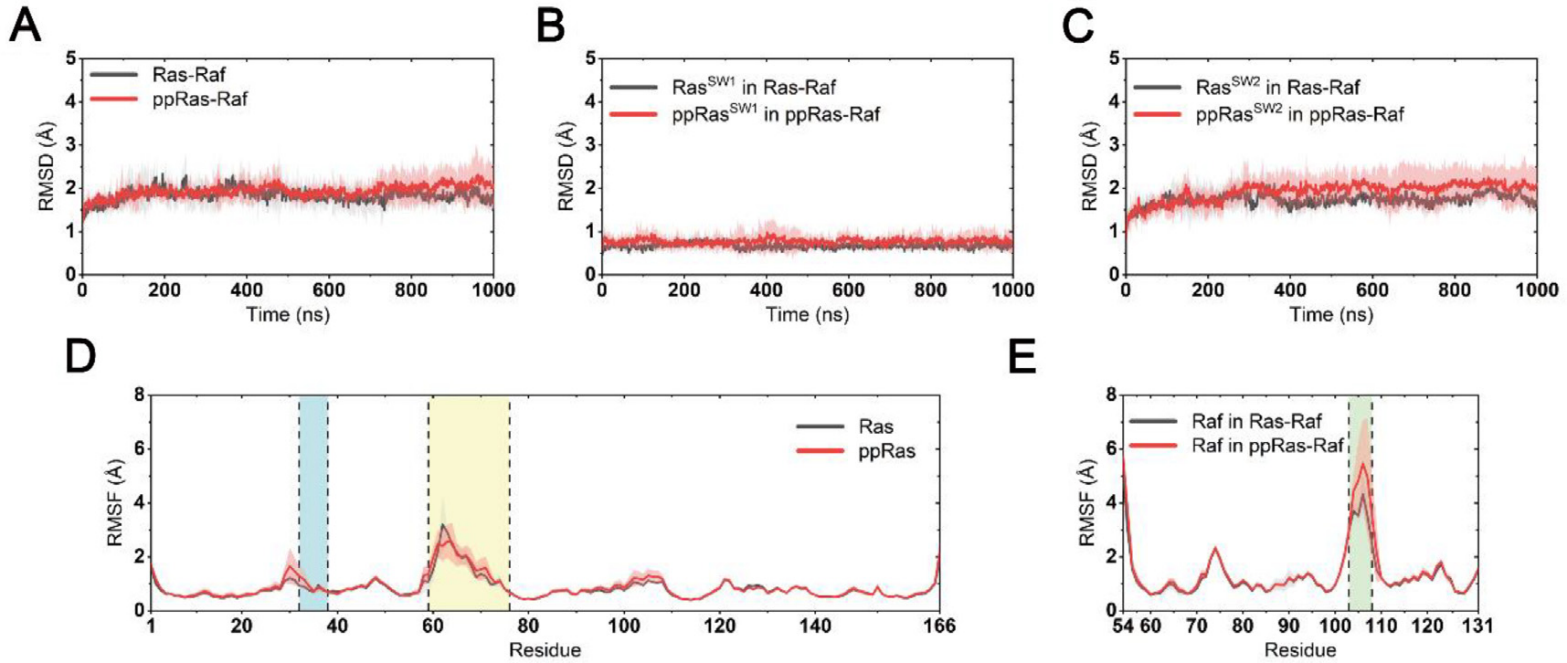
Conformational dynamics of unphosphorylated and phosphorylated Ras in complex with RafRBD. RMSDs of Cα atoms in unphosphorylated (black) and phosphorylated (red) systems within (A) all Ras and RafRBD residues; (B) Ras switch I residues, and (C) Ras switch II residues. Gray and pink transparencies represent the error. (D) RMSF of Ras residues. The two switch regions are marked with blue and yellow backgrounds. (E) RMSF of RafRBD residues. L4 loop region is marked with green background. (For interpretation of the references to colour in this figure legend, the reader is referred to the web version of this article.)

General overview of the structural dynamics of K-Ras4B–RafRBD complexes were presented by RMSD and RMSF analyses. Both systems reached equilibrium after ~200 ns MD simulations (Fig. 8A), and there were no remarkable overall conformational biases between the two systems since their RMSDs were close (1.86 ± 0.28 Å for the unphosphorylated complex and 2.14 ± 0.30 Å for the phosphorylated complex). More specifically, calculations on the RMSDs of the two switch regions suggest that despite the induction of two phosphotyrosines in these two regions, they shared similar backbone conformations in the Ras–RafRBD complexes (Fig. 8B-C). However, in both unphosphorylated and phosphorylated systems the standard deviations of switch II RMSD among all individual replicas were slightly higher than that of switch I, indicating less constraints on the switch II regions. This notion is supported by the RMSF analyses, where in both systems Ras switch II exhibited higher flexibilities (Fig. 8D). Notably, the N-terminal residues of switch I (residues 29–31), which participated in the Ras/Raf interfaces, showed slightly higher RMSFs in the phosphorylated system. This may partially result in weakened Ras–Raf interactions. It also could be noted that phosphorylation induces a mild decrease in the elasticity of the N-termini of the switch II region. However, these residues are away from the interfacial areas between Ras and RafRBD. Due to the lack of structural information on Ras in complex with the full-length Raf kinase domain, whether this slight difference in the conformational dynamics of Ras switch II will affect the interactions between Ras and RafCRD remains unknown.

Next, comparisons of RafRBD binding affinities were carried out from both energetic and structural aspects to explore the effect of phosphorylation on Ras–Raf interactions. As presented in Table 3, we calculated the Ras–RafRBD binding free energies using the MM/PBSA method. The overall $\Delta G_{\mathrm{binding}}$ of unphosphorylated and phosphorylated systems were −84.00 ± 4.93 kcal/mol and −79.31 ± 7.18 kcal/mol, respectively, showing only slight differences. Detailed investigations suggested that although the negatively charged phosphotyrosine in Ras switch I significantly strengthened Ras electrostatic interactions with positively charged alkaline residues on RafRBD, higher solvation energy disfavoring the binding process also occurred, resulting in similar overall binding free energies. In addition, we compared K-Ras4B–RafRBD contacts on the interfacial areas to report the influence of phosphorylation on RafRBD binding affinity. Salt bridges between Ras switch I region and RafRBD are the dominant interacting factors in the interfacial areas [39], [71]. In both of our systems, we observed five pairs of long-lived salt bridges stemming from ionizable residues on the Ras–Raf interface (Fig. S8A). We calculated the probability distributions of inter-residue distances that participated in the formation of salt bridges to reflect the strength of these salt bridges among Ras and Raf (Fig. S8B). In accordance with MM/PBSA results, mild distinctions of distance distributions were found between unphosphorylated and phosphorylated systems, suggesting that no predominant changes in interfacial interactions were observed during our simulations. Taken together, these observations indicated that dual phosphorylation may induce only a slight decrease in Ras–RafRBD binding affinity.

**Table 3:** Binding free energy (kcal/mol) analysis between Ras and RafRBD.*

	Ras-Raf	ppRas-Raf
$\Delta E_{\mathrm{vdw}}$^a^	−65.6703 (4.9437)	−56.9724 (6.6101)
$\Delta E_{\mathrm{ele}}$^b^	−238.8668 (19.1057)	−317.0302 (28.6552)
$\Delta E_{\mathrm{PB}}$^c^	228.1599 (16.8855)	301.3292 (27.2967)
$\Delta E_{\mathrm{nonpolar}}$^d^	−7.6188 (0.4523)	−6.6340 (0.7461)
$\Delta E_{\mathrm{MM}}$^e^	−304.5371 (19.3384)	−374.0026 (31.1789)
$\Delta G_{\mathrm{solv}}$^f^	220.5411 (16.6823)	294.6953 (26.7403)
$\Delta G_{binding}$	−**83.9960 (4.9329)**	−**79.3073 (7.1762)**

*a. The van der Waals component and *b*. the electrostatic component in the change of the total molecular mechanical energy of the Ras–RafRBD complex. *c.* The electrostatic component determined by the Poisson Boltzmann (PB) equation. *d*. The nonpolar component determined by the solvent-accessible surface term in the change of the solvation free energy. *e.* Total molecular mechanical energy change and *f.* total solvation energy change. All numbers in parentheses represent standard deviations.

**Fig. 9:**
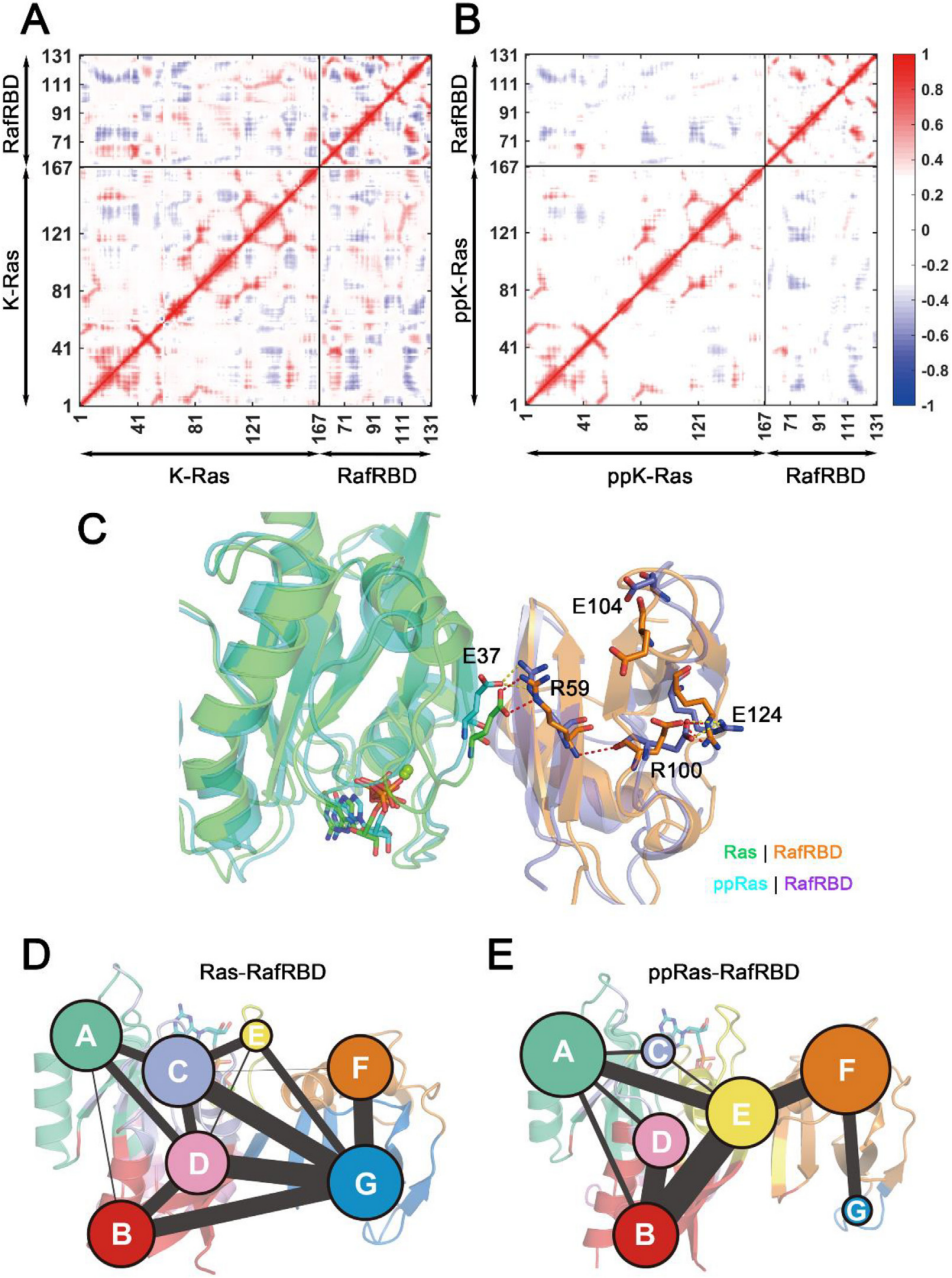
Ras–RafRBD allosteric interactions. DCCMs of (A) unphosphorylated and (B) phosphorylated Ras–RafRBD systems. Positive regions (red) stand for correlated motions, whereas negative regions (blue) represent anti-correlated motions. Correlated motions with absolute values <0.3 were neglected and shown in white. (C) Representative structures of the salt bridge network from Ras residue E37 to RafRBD R100 in unphosphorylated and phosphorylated systems. Salt bridges in the unphosphorylated system are marked with red dashed lines and the same are marked with yellow dashed lines in the phosphorylated system. Map of community network in unphosphorylated (D) and phosphorylated (E) Ras–RafRBD complexes. Areas of the circles represent the numbersof residues in corresponding communities, and the widths of sticks connecting communities represent the intercommunity connections. (For interpretation of the references to colour in this figure legend, the reader is referred to the web version of this article.)

In addition to the interfacial area of Ras–RafRBD complex, we also noted that loop L4 in RafRBD (residues 103–108) showed higher fluctuations upon Ras phosphorylation (Fig. 8E). Previous studies elucidated how the H-Ras Q61L mutation allosterically quenched the flexibility of the L4 loop in RafRBD [39]. Similarly, in our MD simulations dual Y32 and Y64 phosphorylation was found to lead to the destabilization of the remote Raf L4 loop. We then focused concentrated on the exploration of the allosteric pathway from K-Ras4B to the distal Raf L4 loop in both unphosphorylated and phosphorylated systems. Using dynamic cross-correlation matrices (DCCM) calculations, we provide an overview of the inter-residue correlations in the two systems. As shown in Fig. 9A-B, compared to the unphosphorylated complex, the phosphorylated Ras–RafRBD complex exhibited significantly decreased correlated motions among distant residues. Particularly, in the phosphorylated system the correlation of inter-molecular motions among Ras and Raf residues as well as the intra-molecular motions of Raf residues were compellingly weakened, suggesting impaired signal propagation pathways from Ras towards Raf, and also within Raf. In addition, an allosteric pathway that connects the Ras-Raf interface and Raf L4 loop was also disordered in the phosphorylated system (Fig. 9C). Based on an intramolecular salt bridge network, Ras–Raf interactions on the interface propagated through Raf R59 and E124 residues to R100 located at the N-terminus of the L4 loop, which can further rigidify the L4 conformation by interacting with E104 in mutant Ras–Raf complexes [39]. However, salt bridges between Raf R59 and E124 were predominantly weakened in the phosphorylated system, interrupting the propagation pathway. This could also explain the loss of allosteric regulation between Ras and RafRBD.

Community network analysis and allosteric pathway analysis were carried out to systematically investigate the allosteric network in Ras–RafRBD complexes. Residues within a 4.5 Å cut-off distance for at least 75% of the time during MD simulations were categorized into the same community, which could be considered as a congenerous unit within the complex [50]. As shown in Fig. 9D-E, there were seven communities in both the unphosphorylated and phosphorylated systems. Each community is represented by a colored circle, whose area is in proportion to the number of residues it contains, and is superimposed on the 2D structure of the Ras–Raf complex to represent the relative positions with adjacent communities. In general, the residual components of each community were similar in the two systems, and the detailed configurations of the communities are shown in Fig. S9. However, communities on the Ras–Raf interface and within RafRBD exhibited significantly different constitutions. In the unphosphorylated system the Ras–Raf interface mainly consisted of Community E, F, and G, while in the phosphorylated complex Community G, which contains Raf L4 residues was torn apart from interfacial communities. More importantly, in the unphosphorylated complex residues that participated in the above-mentioned salt-bridge propagation pathway (Fig. 9C) were all incorporated in Community G, suggesting that they were in close proximity throughout the simulations. On the contrary, the allosteric communication pathway from the interface through Raf intermediate residues to the remote Raf L4 loop, was separated into three different communities in the phosphorylated system, indicating less synergistic cooperation among the residues along the Ras-Raf allosteric pathway.

On the other hand, structural information flow between the communities was calculated based on graph theory and topology. The edge connectivity among communities is defined by the number of shortest paths that pass through the edging nodes, and in Fig. 9D-E the strength of intercommunity connections is reflected by the width of the sticks. In the unphosphorylated complex, it could be noted that four communities consisting of Ras residues, including Community B, C, D and E, were in direct and strong connections with Community G which contains Raf L4 loop residues (Fig. 9D). In contrast, all direct connections between Ras communities and Community G were lost in the phosphorylated system (Fig. 9E). Additionally, by calculating the optimal and suboptimal pathways that link the Ras/RafRBD interface and Raf L4, we revealed the potential allosteric relationships between the chosen residues in the two systems. As listed in Table S3, Ras interfacial residues in the unphosphorylated system displayed shorter lengths of the optimal pathways, less involved residues and larger numbers of suboptimal pathways towards Raf L4, indicating extensive and strong allosteric relationships between the two regions in the unphosphorylated system relative to the phosphorylated system. These results, together with DCCM analyses, collectively demonstrated that dual phosphorylation interdicted allosteric regulations from K-Ras4B towards RafRBD.

## 4 Discussion

Ras proteins, as molecular switches, control a myriad of intracellular signaling pathways. Oncogenic mutations disrupt the equilibrium of the ‘on’ and ‘off’ status of Ras, either by Ras GTP/GDP interconversion through GTPase cycle, or by impacting Ras–effector interactions, leading to abnormal signaling for cell growth, proliferation, and differentiation [1], [2], [3], [72]. In addition to mutations, recent studies have shown that Ras phosphorylation is also involved in the regulation of Ras signaling pathways [34], [73]. Here, using MD simulations, we provided structural insights into how K-Ras4B dual Y32 and Y64 phosphorylation regulated by Src/SHP2 could influence its activities, thereby motivating progress in targeting Ras phosphorylation for cancer drug discovery.

By comparing the on conformational dynamics of unphosphorylated and phosphorylated K-Ras4B in both GTP- and GDP-bound states, as well as their interactions with regulatory proteins GAP/SOS or effector Raf, we have revealed the mechanism by which dual phosphorylation disrupts the GTPase cycle and impairs downstream Raf signaling. For both GTP- and GDP-bound states of Ras, the most predominant structural differences induced by phosphorylation was found to be in the nucleotide-binding pocket. The intrinsic hydrolysis of GTP is catalyzed by the conjugated arrangements of Ras G13, Y32, T35 and Q61, which neutralize the negative charges on GTP and activate the nucleophilicity of the adjacent water molecule (Fig. 3A). Previous studies could not reach a consensus on how the introduction of a phosphate group on Ras Y32 would influence its intrinsic hydrolysis rate [34], [73]. Our observations indicated that upon phosphorylation, the catalytic conformation for GTP hydrolysis was distorted, and therefore Ras activity as a GTPase is weakened. Specifically, the most significant distinction occurred in the side-chain orientation of Y32. In the phosphorylated system, pY32 adopted an outward orientation pointing away from the GTP γ-phosphate. Since close contacts between Y32 and GTP are favorable for effector binding [55], our observations indicated decreased signal conductivity of the phosphorylated Ras. Likewise, in the phosphorylated GDP-bound Ras, the switch I region moved away from GDP, thus expanding the nucleotide binding pocket. In general, it could be concluded from both GTP- and GDP-bound K-Ras4B systems that upon phosphorylation, the two switch regions tend to deviate from the nucleotide phosphate group, resulting in the stabilization of inactive states (e.g. inactive state 1) and the loss of intrinsic catalytic activity of Ras-GTP, as well as the expansion of nucleotide pocket in Ras-GDP. Both the electrostatic effect of negatively charged phospho-tyrosine groups and the steric hindrance effect derived from the bulky phosphate groups contribute to the conformational transitions on the two switch regions. These factors collectively resulted in the impairment of GTP hydrolysis activity and the accelerated GDP disassociation rate for phosphorylated K-Ras4B, in accordance with previous experimental conclusions [34].

Moreover, phosphorylation not only altered Ras conformations in the free forms, but also perturbed Ras PPIs. From both energetic and conformational aspects, we have revealed phosphorylation effects on Ras interactions with its regulators GAP and SOS as well as its effector Raf. In terms of binding energies, our calculations suggest that phosphorylated Ras exhibited weakened interactions with cell signaling proteins, especially with SOS (Table 1, Table 2, Table 3). Structural insights into the interfacial electrostatic interactions (salt bridges) and hydrogen bonds have further confirmed the impaired polar interactions between Ras and SOS induced by Ras phosphorylation (Fig. 7, Table S1&S2). In addition to changes in binding affinities, the catalytic sites of Ras regulators that greatly facilitate Ras GTP hydrolysis or GDP exchange were also disordered. In the unphosphorylated Ras–GAP complex, GAP arginine finger R789 protruded into the GTP pocket and mimicked the hydrolysis-active conformation in free Ras-GTP to accelerate GTP hydrolysis. However, in the phosphorylated system, GAP R789 exhibited higher fluctuations and could not stabilize the catalytic location, thereby regulating its function on Ras (Fig. 5). The relative size of the GDP binding pocket between unphosphorylated and phosphorylated Ras–SOS complexes was reversed compared to the free Ras-GDP, which favors GDP disassociation in the unphosphorylated Ras–SOS system. These evidences together clarified the underlying mechanisms for the loss of functions of GAP and SOS towards phosphorylated K-Ras4B [34], [73]. During investigation of Ras–Raf interactions, we observed blocked intercommunications between K-Ras4B and Raf due to phosphorylation (Fig. 9). Particularly, from community analyses and suboptimal pathway comparisons, we found that the allosteric propagation pathway from the Ras/Raf interface to the Raf distal L4 loop was interrupted upon phosphorylation. Since the L4 region of RafRBD is considered to play a role in Raf kinase activation [39], [74], Ras phosphorylation may affect its functions in an allosteric manner.

In view of the crucial role played by Ras in cancer pathogenesis [3], the development of Ras inhibitors has been the major focus over the past three decades. However, until now, only few Ras direct inhibitors have been discovered with good potency and clinical application value [75]. This is largely due to the obstacles in recognizing druggable pockets on the smooth surface of Ras. Some alternative strategies for anti-Ras therapies focused on targeting Ras PPIs with regulatory proteins (e.g., SOS and GAP) or with its downstream effectors (e.g., Raf) [14], [76], [77], [78], [79]. However, the structural basis for Ras–regulator/effector interactions need to be further investigated for structure-based drug design. Our explorations of the conformational outcomes as well as the PPI alterations induced by phosphorylation have highlighted new aspects of cancer drug design by targeting phosphorylated Ras as well as its interactions with other cell signaling proteins. Ras-GTP exists in rapidly interconverting active and inactive sub-states [4], [22], and except for their distinctions in signaling functions, particular druggable pockets occur in certain substates [80], [81], [82], [24]. Here, an inactive state 1 conformation of GTP-bound K-Ras4B was only discovered in the phosphorylated system (Fig. 2). Analyses of the representative structures detected a potential druggable pocket, which is mainly composed of switch II regions (Fig. S3). This pocket resembles the previously reported pocket in the GTP-bound H-Ras, with an even higher druggability score indexed by fpocket software. This inspires future research aimed at the development of small molecules that directly inhibit phosphorylated Ras.

## Author contributions

S.L. conceived and supervised the project; S.L., Y.L., Q.F., Y.W., J.Z., and N.D. designed computational simulations and revised the manuscript; Y.W., D.J., C.L., and Y.C. performed the simulations, analyzed the results and drafted the manuscript; Y.Q., X.L., M.L., and J.P. revised the manuscript; All authors discussed the results and reviewed the manuscript.

## Conflicts of interest statement

The authors declare no conflict of interest.
