# Supplementary material for: Mechanistic insights into the effect of phosphorylation on Ras conformational dynamics and its interactions with cell signaling proteins
Source: Comput Struct Biotechnol J. 2021 Feb 9;19:1184–99. doi: 10.1016/j.csbj.2021.01.044 (PMC7902900; doi:10.1016/j.csbj.2021.01.044)
Supplement: Supplementary data 1 [file mmc1.docx]

**Supplementary Information**

**Mechanistic insights into the effect of phosphorylation on Ras conformational dynamics and its interactions with cell signaling proteins**

Yuanhao Wang^1,§^, Dong Ji^2,§^, Chaoyu Lei^1^, Yingfei Chen^1^, Yuran Qiu^1^, Xinyi Li^1^, Mingyu Li^1^, Duan Ni^1,3^, Jun Pu^4^, Jian Zhang^1,5^, Qiang Fu^6,*^, Yaqin Liu^5,*^, Shaoyong Lu^1,5,*^

^1^Department of Pathophysiology, Key Laboratory of Cell Differentiation and Apoptosis of Chinese Ministry of Education, Shanghai Jiao Tong University, School of Medicine, Shanghai 200025, China

^2^Department of Anesthesiology, Changhai Hospital, The Second Military Medical University, Shanghai 200433, China

^3^The Charles Perkins Centre, University of Sydney, Sydney, NSW 2006, Australia

^4^Department of Cardiology, Renji Hospital, Shanghai Jiao Tong University, School of Medicine, Shanghai 200120, China

^5^Medicinal Chemistry and Bioinformatics Centre, Shanghai Jiao Tong University, School of Medicine, Shanghai 200025, China

^6^Department of Orthopedics, Shanghai General Hospital, Shanghai Jiao Tong University, School of Medicine, Shanghai 200080, China

^§^These authors contributed equally to this work.

*To whom correspondence should be addressed:

Dr. Shaoyong Lu; E-mail: [lushaoyong@yeah.net](mailto:lushaoyong@yeah.net)

Dr. Yaqin Liu; E-mail: [liuyaqing7811@126.com](mailto:liuyaqing7811@126.com)

Dr. Qiang Fu; E-mail: johson.f@163.com


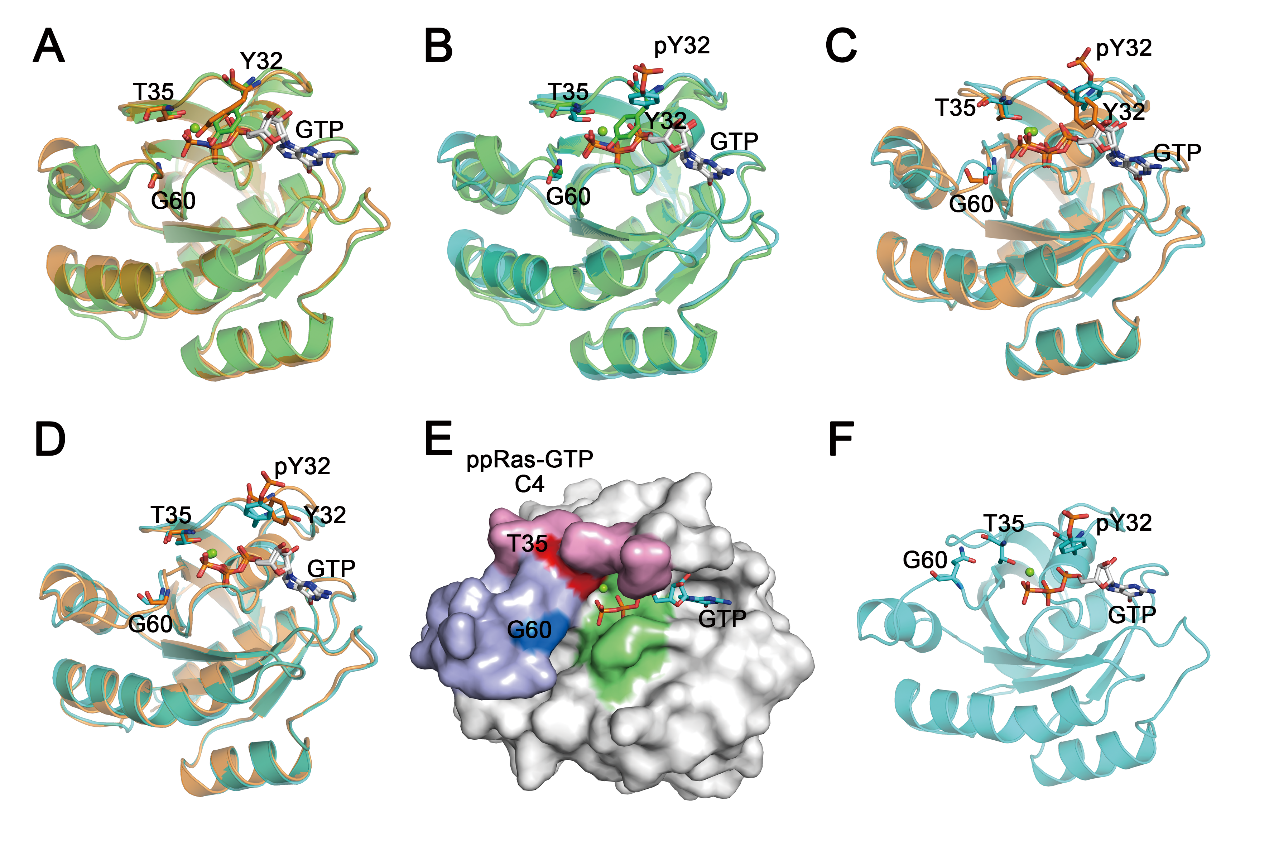


**Figure S1.** Representative structures of conformations in Ras-GTP free-energy landscape basins C1-C4. (A) Representative structure of unphosphorylated K-Ras-GTP C1 (orange) superimposed on active-form H-Ras-GTP (green) (PDB ID 3K8Y). (B) Representative structure of phosphorylated K-Ras-GTP C1 (cyan) superimposed on active-form H-Ras-GTP (green). (C) Representative structures of unphosphorylated K-Ras-GTP C2 (orange) and phosphorylated K-Ras-GTP C2 (cyan). (D) Representative structures of unphosphorylated K-Ras-GTP C3 (orange) and phosphorylated K-Ras-GTP C3 (cyan). (E) Representative structure of phosphorylated K-Ras-GTP C4. Ras P-loop, Switch I and Switch II regions are colored as lime, pink and light blue, respectively. Surface of T35 and G60 residues are colored as red and marine. (F) Cartoon representation of the representative structure of phosphorylated K-Ras-GTP C4.


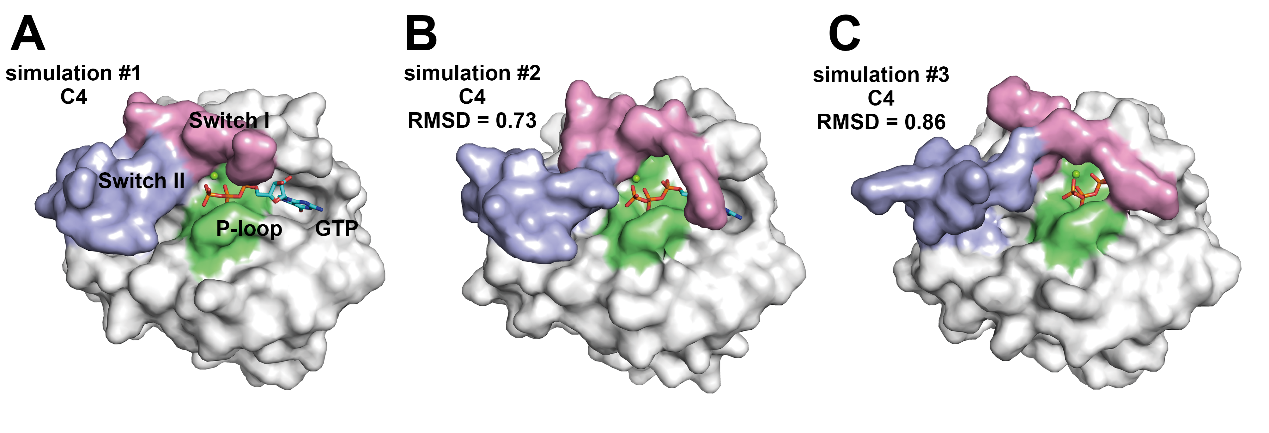


**Figure S2.** Representative structures of conformation C4 of dual phosphorylated GTP-bound K-Ras4B in three independent runs. The RMSD of backbone C_α_ atoms of C4 representative structures in other two independent runs relative to the first run is 0.73 and 0.86, respectively.


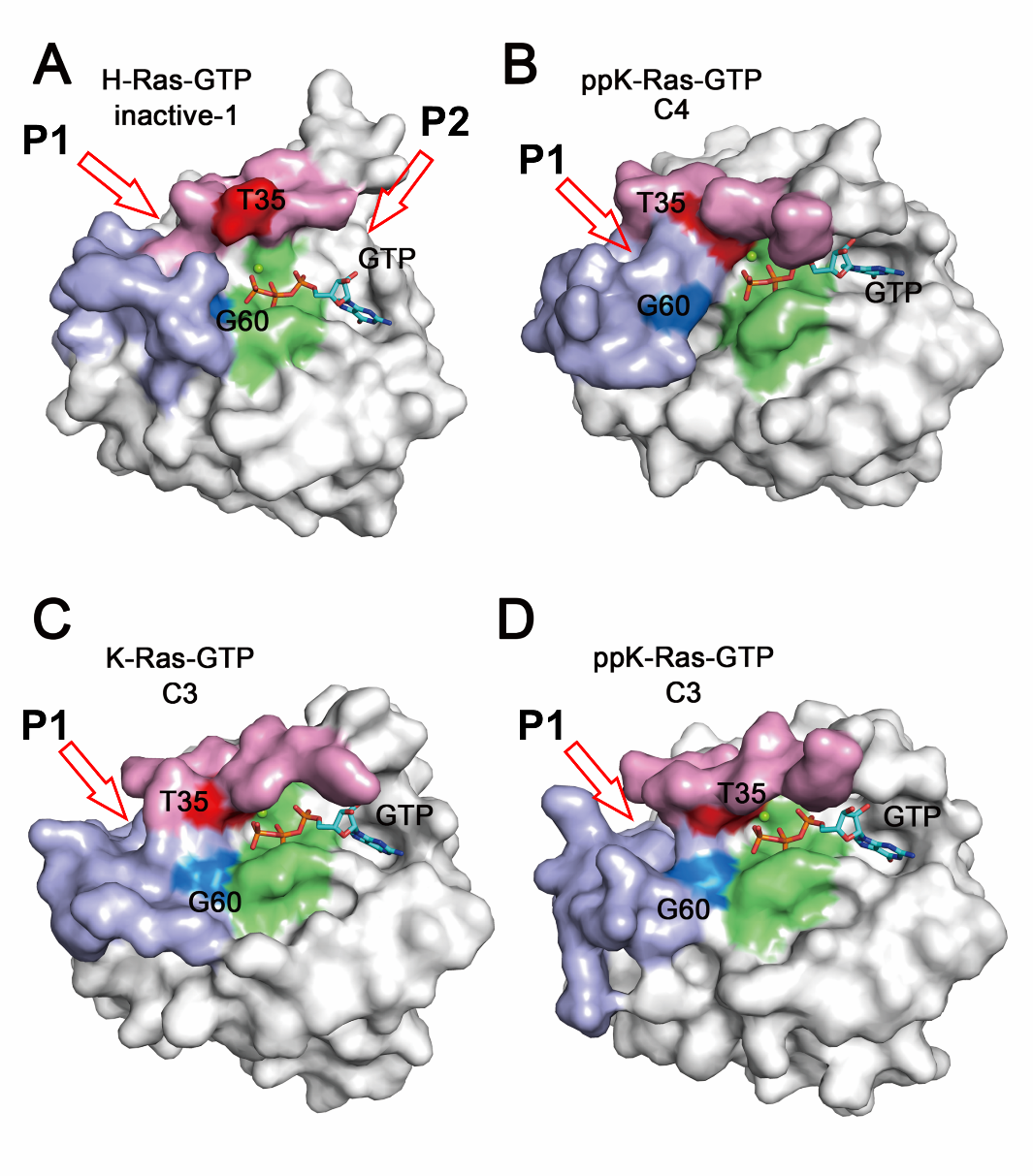


**Figure S3.** Potential druggable pockets on Ras surface. (A) Crystal structure of inactive 1 state H-Ras^WT^-GTP (PDB ID 4EFL). (B) Representative structure of phosphorylated K-Ras-GTP C4. (C) Representative structure of unphosphorylated K-Ras-GTP C3. (D) Representative structure of phosphorylated Ras-GTP C3. Ras P-loop, Switch I and Switch II regions are colored as lime, pink and light blue, respectively.


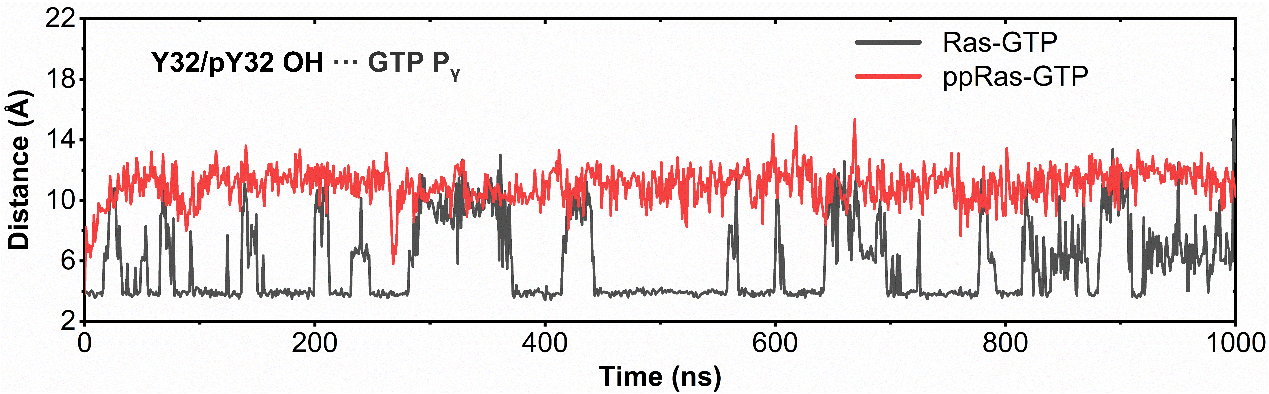


**Figure S4**. Distance between Y32/pY32 OH atom and GTP P_γ_ versus time in GTP-bound K-Ras4B.


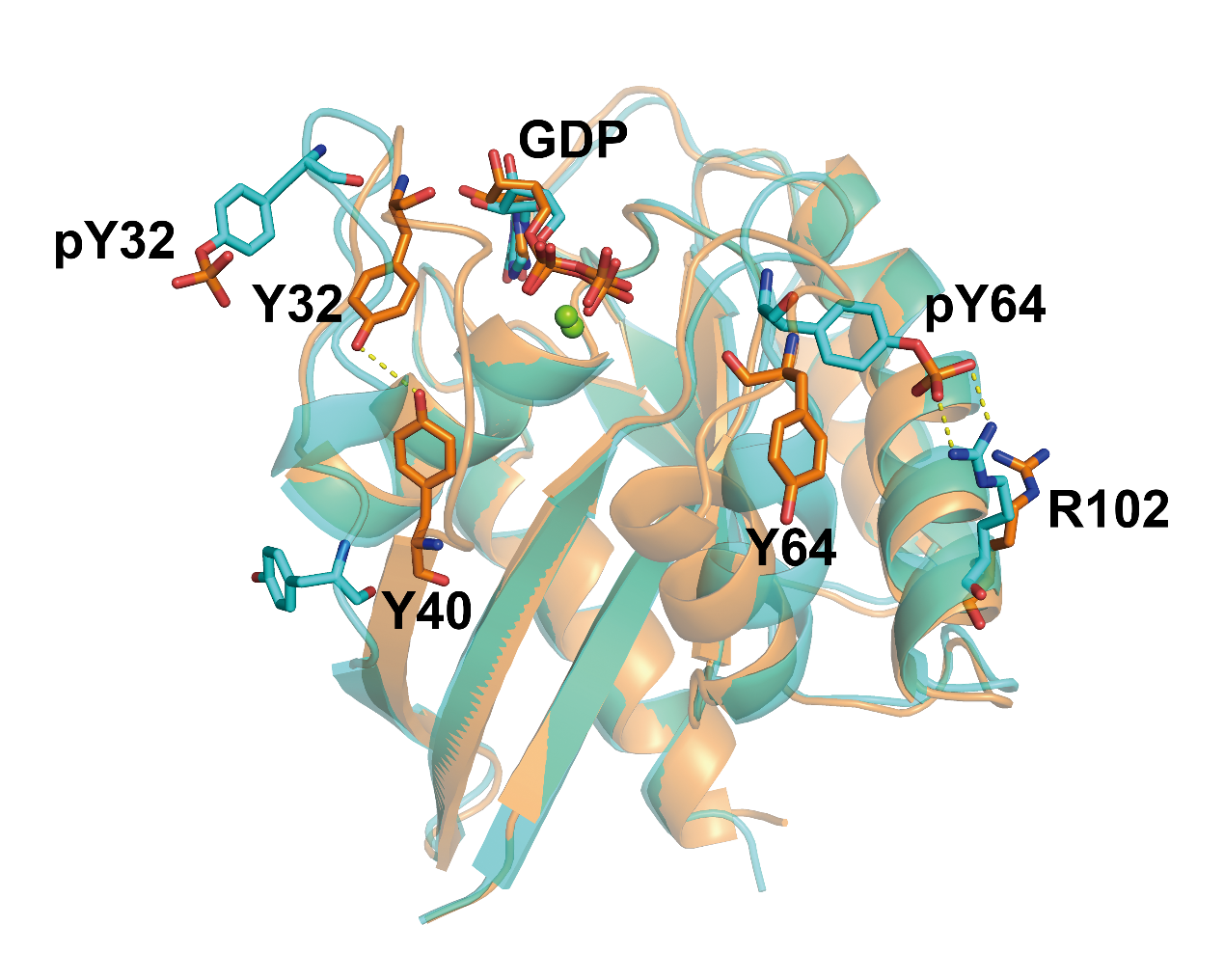


**Figure S5**. Representative structure of the unphosphorylated (orange) and phosphorylated GDP-bound Ras. Intra-molecular hydrogen bonds and salt bridges is deciphered by yellow dashed lines.





**Figure S6.** Changes in angle of Ras Q61 NE2 atom to Ras Q61 OE1 atom to GTP P_γ_ atom over time in the unphosphorylated and phosphorylated Ras−GAP complex. The high volatility in the phosphorylated system indicates a lack of stabilization of the Ras Q61 side chain for GTP hydrolysis.


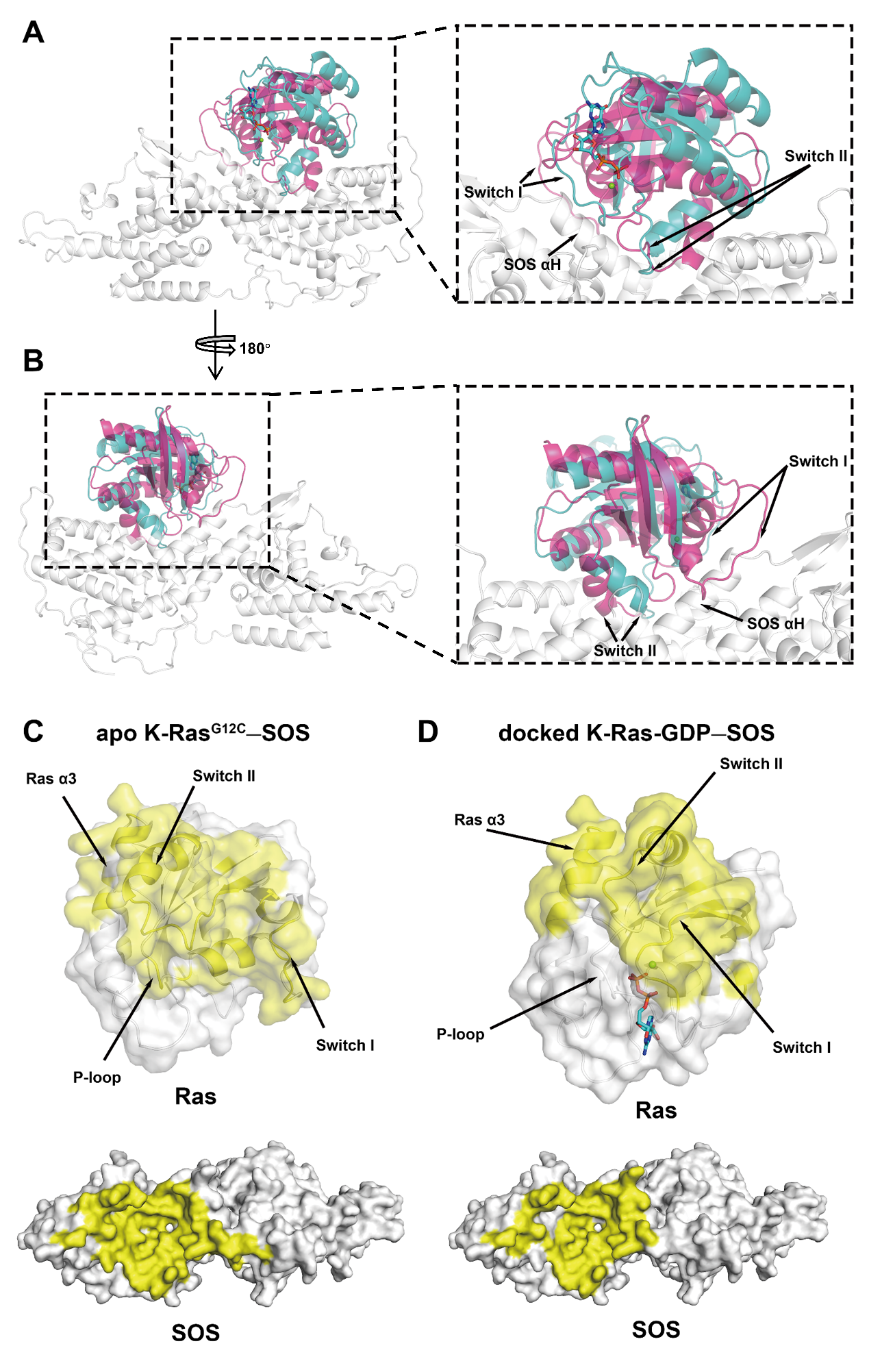


**Figure S7.** Alignment of crystal apo K-Ras^G12C^−SOS (PDB ID 6EPL) and the docked complex of GDP-bound K-Ras to SOS. (A) Front and (B) back view of crystal apo K-Ras−SOS complex (magenta) and the docked complex of K-Ras-GDP−SOS (cyan). Interfacial residues of (C) the apo K-Ras−SOS complex and (D) the docked K-Ras-GDP−SOS complex is shown in yellow.


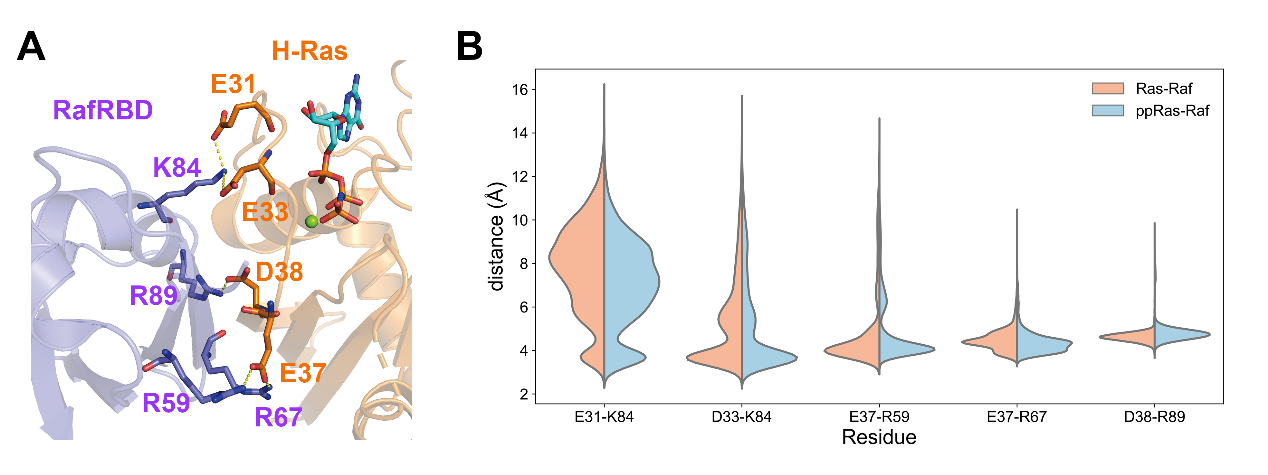


**Figure S8.** K-Ras4B−RafRBD interface. (A) Interfacial residues that form salt bridges between K-Ras4B and RafRBD (reference structure: H-Ras in complex with RafRBD, PDB ID 4G0N). (B) Probability distributions of inter-residue distances participate in the interfacial salt bridges. Distances are measured among Glu CD atoms, Asp CG atoms, Lys NZ atoms and Arg CZ atoms to reflect the strength of the salt bridges.


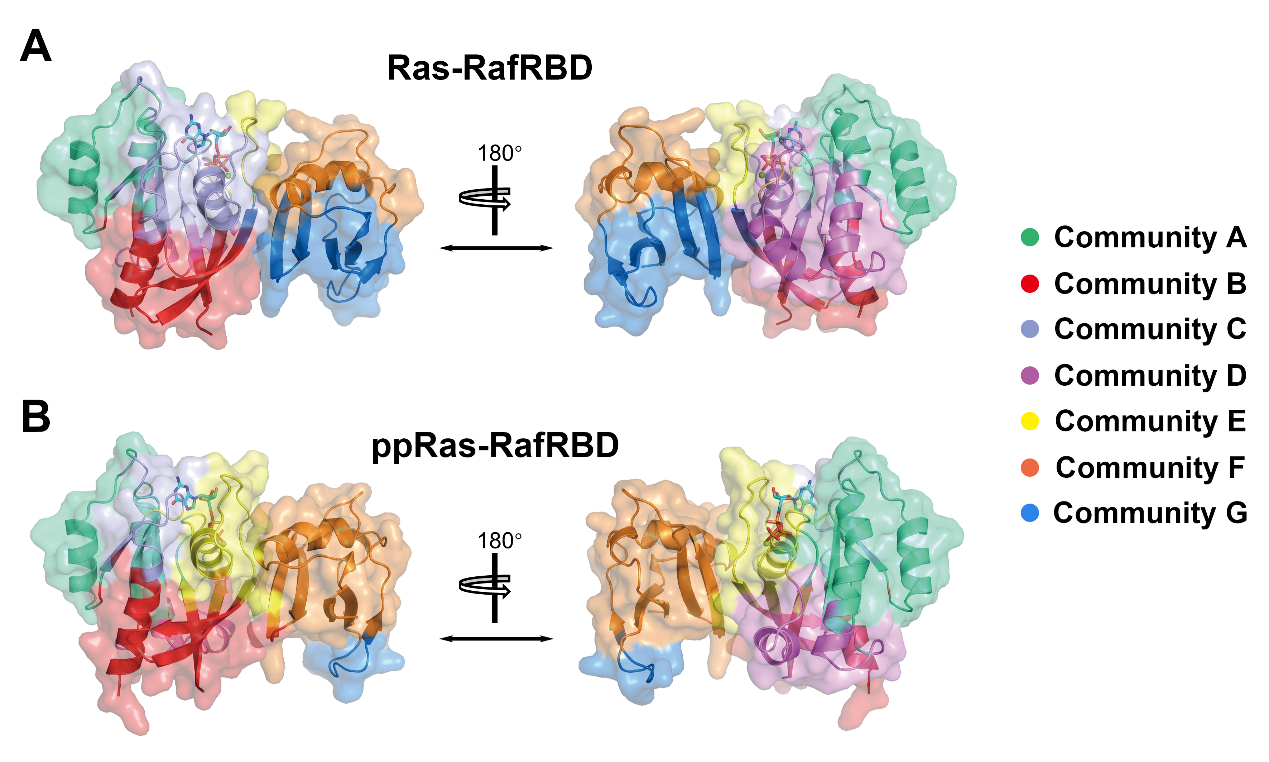


**Figure S9.** Community configurations of (A) unphosphorylated and (B) phosphorylated K-Ras4B−RafRBD complexes.

**Table S1.** Inter-molecular hydrogen-bonds on the K-Ras4B−SOS interface

| No. | **Ras**  **atom** | **SOS**  **atom** | **Length**  **(Å)** | **ppRas**  **atom** | **SOS**  **atom** | **Length**  **(Å)** |
| --- | --- | --- | --- | --- | --- | --- |
| 1 | Thr35 OG1 | Gly943 H | 2.32 | PTR64 O1P | Lys1003 HZ3 | 2.29 |
| 2 | Asp38 OD2 | Lys939 HZ3 | 1.86 | PTR64 O2P | Lys1003 HZ1 | 2.44 |
| 3 | Asp105 OD1 | Arg1019 HH21 | 1.78 | Asp105 OD1 | Arg1019 HH21 | 1.73 |
| 4 | Asp105 OD2 | Arg885 HE | 1.94 | Asp105 OD2 | Arg885 HE | 1.76 |
| 5 | Asp105 OD2 | Arg885 HH21 | 2.15 | Asp105 OD2 | Arg885 HH21 | 1.76 |
| 6 | Glu37 H | Glu942 OE1 | 1.96 |  |  |  |
| 7 | Arg41 HH12 | Glu909 OE1 | 1.94 |  |  |  |
| 8 | Arg41 HH22 | Glu909 OE1 | 1.92 |  |  |  |
| 9 | Arg73 HE | Asn879 O | 1.88 |  |  |  |
| 10 | Arg73 HH21 | Asn879 O | 1.90 |  |  |  |
| 11 | Arg102 HH12 | Asp1007 OD1 | 1.62 |  |  |  |

**Table S2.** Inter-molecular salt bridges on the K-Ras4B−SOS interface

| No. | **Ras**  **atom** | **SOS**  **atom** | **Length (Å)** | **ppRas**  **atom** | **SOS**  **atom** | **Length (Å)** |
| --- | --- | --- | --- | --- | --- | --- |
| 1 | Asp38 OD2 | Lys939 NZ | 2.77 | Asp38 OD1 | Lys939 NZ | 2.84 |
| 2 | Asp105 OD1 | Arg885 NE | 3.66 | Asp38 OD2 | Lys939 NZ | 2.65 |
| 3 | Asp105 OD1 | Arg1019 NE | 2.99 | Glu62 OE1 | Lys1003 NZ | 2.77 |
| 4 | Asp105 OD1 | Arg1019 NH2 | 2.76 | Glu62 OE2 | Lys1003 NZ | 2.82 |
| 5 | Asp105 OD2 | Arg885 NE | 2.80 | Asp105 OD1 | Arg1019 NE | 2.82 |
| 6 | Asp105 OD2 | Arg885 NH2 | 2.99 | Asp105 OD1 | Arg1019 NH2 | 2.69 |
| 7 | Asp105 OD2 | Arg1019 NE | 2.98 | Asp105 OD2 | Arg885 NH2 | 2.71 |
| 8 | Arg41 NE | Asp910 OD1 | 3.65 | Asp105 OD2 | Arg885 NE | 2.73 |
| 9 | Arg41 NH1 | Glu909 OE1 | 2.87 | Asp105 OD2 | Arg1019 NE | 2.88 |
| 10 | Arg41 NH2 | Glu909 OE1 | 2.82 | Asp105 OD2 | Arg1019 NH2 | 3.86 |
| 11 | Arg41 NH2 | Glu909 OE2 | 3.44 | Arg41 NH1 | Asp910 OD2 | 2.91 |
| 12 | Arg102 NH1 | Asp1007 OD1 | 2.61 | Arg41 NH2 | Asp910 OD1 | 3.76 |
| 13 | Arg102 NH1 | Asp1007 OD2 | 3.98 | Arg41 NH2 | Asp910 OD2 | 3.43 |
| 14 | Arg102 NH2 | Asp1007 OD1 | 2.83 |  |  |  |
| 15 | Arg102 NH2 | Asp1007 OD2 | 3.10 |  |  |  |

**Table S3.** Allosteric pathway analysis between K-Ras4B/RafRBD interface to RafRBD L4 loop

| **Pathway** | **Length (Å)***^a^* | | **Residue***^b^* | | **Subopt*^c^*** | |
| --- | --- | --- | --- | --- | --- | --- |
|  | Ras−Raf | ppRas−Raf | Ras−Raf | ppRas−Raf | Ras−Raf | ppRas−Raf |
| Ras E31 –  Raf E104 | 331 | 527 | 9 | 12 | 11 | 5 |
| Ras E33 –  Raf E104 | 294 | 482 | 8 | 11 | 11 | 5 |
| Ras E37 –  Raf E104 | 218 | 410 | 6 | 9 | 5 | 3 |
| Ras D38 –  Raf E104 | 226 | 405 | 7 | 9 | 7 | 3 |

**a*. Length of the shortest pathways from Ras/RafRBD interface towards Raf L4 loop. *b.* Numbers of residues involved in the optimal pathways. *c*. Numbers of the suboptimal pathways from Ras/RafRBD interface towards Raf L4 loop.
